# Red mite (*Panonychus citri*) attack amplifies citrus rootstock-driven responses in physiological and biochemical traits, VOC emission, and expression of defence-related genes in mandarin scions

**DOI:** 10.3389/fpls.2025.1645535

**Published:** 2025-09-04

**Authors:** Tommy Rioja, Karina B. Ruiz, Ricardo Ceballos

**Affiliations:** ^1^ Departamento de Recursos Ambientales, Facultad de Ciencias Agronómicas, Universidad de Tarapacá, Arica, Chile; ^2^ Química y Farmacia, Facultad de Ciencias de la Salud, Universidad Arturo Prat, Iquique, Chile; ^3^ Chemical Ecology Laboratory, Instituto de Investigaciones Agropecuarias, Instituto de Investigaciones Agropecuarias (INIA) Quilamapu, Chillán, Chile

**Keywords:** biotic stress marker genes, fruit trees, plant-insect interaction, scion/rootstock interaction, volatile organic compounds, salicylic acid

## Abstract

Citriculture faces significant constraints in expanding into new environments and agroecological zones. Grafting onto tolerant rootstocks has helped overcome some of these limitations, enabling cultivation under diverse conditions. Nevertheless, citrus production remains vulnerable to multiple abiotic and biotic stressors, among which red mite (*Panonychus citri*) herbivory can markedly reduce yield and fruit quality. While rootstocks are known to influence scion physiology and defence capacity, their specific role in modulating responses to pest attack is still poorly understood. To address this, we evaluated 18-month-old ‘W. Murcott’ mandarin grafted onto four citrus rootstocks (‘Macrophylla’, ‘C35’, ‘Citrumelo’, ‘Carrizo citrange’) under semi-field conditions, infested or not with *P. citri*. After seven days of infestation (100–160 eggs/leaf), we quantified stress markers (malondialdehyde, proline, salicylic acid), physiological parameters, primary and secondary metabolites, volatile organic compounds (VOCs), and defence-related gene expression. Rootstocks significantly modulated constitutive and inducible responses. ‘Citrumelo’ and ‘Carrizo’ showed the lowest MDA accumulation and strongest induction of SA, *PR5*, and *GLR* transcripts, coupled with increased emission of herbivory-induced plant volatiles (HIPVs, e.g., β-pinene, methyl salicylate, β-ocimene). ‘Macrophylla’ exhibited limited changes, whereas ‘C35’ displayed high MDA content and *PITY1* induction, suggesting greater oxidative stress. Photosynthetic pigments declined across all combinations after infestation, while soluble sugars and flavonoids decreased in susceptible rootstocks. VOC profiles shifted both qualitatively and quantitatively in a rootstock-dependent manner. These results show that *P. citri* herbivory can amplify rootstock-driven differences in physiological, biochemical, and molecular traits, providing a basis for further studies on the role of rootstock–scion interactions in citrus resistance to mite attack.

## 1 Introduction

Citrus is one of the most important fruit trees worldwide, covering 10.55 million hectares and yielding over 169.38 million tons during 2023 (FAOSTAT, 2025). Among citrus species, mandarins, clementines and tangerines rank second in terms of productive importance, with a combined production of 52,556,927 tons (FAOSTAT, 2025). In Chile, mandarins are primarily cultivated in the north-central regions, spanning from the extremely arid climate of Arica y Parinacota (Azapa Valley, 18°31’ S, 70°10’ W) and Atacama (27°22’ S, 70°19’ W) to the Mediterranean conditions of central Chile (34°22’ S, 71°07’ W), with a total of 12,405.3 hectares (ODEPA, 2025). However, its productivity and that of other Citrus species can be significantly affected by both abiotic and biotic factors, which have been further exacerbated by climate change (Syvertsen and García-Sanchez, 2014; Agut et al., 2016; Nawaz et al., 2021).

Grafting techniques have enabled cultivation of fruit trees in soil-limiting conditions through the use of tolerant and resistant rootstocks (Rasool et al., 2020). These rootstocks can positively influence various characteristics of the scions at the molecular and physiological levels, including vigour, organoleptic fruit quality, yield, and nutrient uptake, among other agronomic traits (Agusti et al., 2020; He et al., 2020; Zhou et al., 2022). It has been reported that rootstocks can confer tolerance to diseases and pests (Agut et al., 2015; Jones and Killiny, 2021; Guarino et al., 2022; Alfaro-Quezada et al., 2023). Hence, rootstocks may significantly impact on features and products of the scion’s primary and secondary metabolism.

It is worth noting that several published studies have investigated the physiological and biochemical parameters of citrus cultivars (Simpson et al., 2014; Arjona-López et al., 2023). However, these studies typically involved non-grafted plants or different cultivars grafted onto the same rootstock under abiotic stress conditions (Long et al., 2017; Huang et al., 2020). Less is known, however, about the effect of citrus rootstocks on scions attacked by pests.

Plants detect herbivores through elicitors/effectors known as damage-associated molecular patterns (DAMPs) and herbivore-associated molecular patterns (HAMPs) (Erb et al., 2012), which activate the production of oxidative molecules, defence-related phytohormones, and expression of genes (Erb et al., 2012; Mishra et al., 2024; Sheri et al., 2023). In addition, plants emit volatile organic compounds (VOCs), specifically herbivory-induced plant volatiles (HIPVs; Erb et al., 2015; Ali et al., 2023), which act as indirect defences agents (Alsabte et al., 2022; Zhou and Jander, 2022) by attracting predators and parasitoids, establishing tri-trophic interactions (Erb et al., 2010; Abdala-Roberts et al., 2019). HIPV emissions can vary depending on Citrus rootstock, as observed in ‘Sugar Belle’ hybrid mandarin scions infested with *Diaphorina citri* (Hemiptera: Liviidae) (Jones and Killiny, 2021).

Biochemical and physiological traits, and several growth attributes have been helpful in identifying plant tolerance against pests (Mitchell et al., 2016). Proline and other amino acids increased in vine (*Vinis vinifera* L.), wheat (*Triticum aestivum* L.), and potato (*Solanum tuberosum* L.) plants infected by pathogens (Anzano et al., 2022); likewise, malondialdehyde (MDA) content, a key bioindicator of plant cell membrane lipid peroxidation, plays a role in plant response to herbivory (Morales and Munné-Bosch, 2019). Similarly, soluble sugars, proteins, and antioxidant molecules are vital for plant development, with critical functions in their defence mechanisms against biotic stresses (Anzano et al., 2022; Hayat et al., 2022; Sheri et al., 2023). Also, polyphenols are pivotal in plant defence mechanisms, acting as crucial deterrents against biotic threats, including herbivores, and serving as protectors against abiotic stresses (Singh et al., 2021).

The main player in plants infested with mites, aphids, and whiteflies is salicylic acid (SA; Mishra et al., 2024), but ethylene (ET), abscisic acid (ABA) and jasmonate (JA) also modulate the expression of defence-related genes (Erb et al., 2010; Li et al., 2019; Yu et al., 2021). In sour orange plants infested with *T. urticae*, *EIN3*, an ET- related transcription factor (TF), and *ABA4*, an ABA biosynthesis-related gene (North et al., 2007), were involved in defence pathways (Agut et al., 2014). SA marker gene*PR-5* (*Pathogenesis Related-5*) is induced early, followed by JA-related *PR-3 (Pathogenesis Related-3)*. *PI Citrus TYPE 1* (*PITY1*) is a proposed infestation marker *glutamate receptor-like* genes (*GLR*) that act as non-specific amino acid sensors in plant defence signalling pathways (Yan et al., 2024).

*Panonychus citri* (McGregor) (citrus red mite; Acari: Tetranychidae), is a major foliar pest of *Citrus* (Zanardi et al., 2015), feeding on the adaxial leaf surface by extracting cell contents, such as chloroplasts (Hoy, 2011) depositing its eggs there. This behavior affects citrus varieties at morphological, physiological, and molecular levels (Agut et al., 2014, 2016), reducing photosynthesis, stomatal conductance, and transpiration, as shown in *Jatropha curcas* plants (Hsu et al., 2015), unlike mite-tolerant varieties. Citrus rootstocks may mitigate infestation effects, making it essential to explore their role in enhancing scion resistance to phytophagous mites (Agut et al., 2014).

This research evaluates four different citrus rootstocks on the commercial mandarin ‘W. Murcott’ when scions were infected by *P. citri* under semi-field conditions. To this purpose, stress markers together with several physiological traits, VOCs, and expression of defence-related genes were analyzed. Our results contribute to refining nursery protocols and identifying optimal scion/rootstock interactions in young citrus plants.

## 2 Materials and methods

### 2.1 Plant material and growth conditions

The study was conducted during the summer of 2022, in 18-month-old mandarin ‘W. Murcott’ (*Citrus reticulata* Blanco) plants at Huayquique, Chile (20° 16’ S; 70° 07’ W; 28 m. a.s.l.). Each scion was grafted onto one of four different rootstocks: ‘Macrophylla’ (MA), ‘C35’ (C35), ‘Citrumelo’ (CI), and ‘Carrizo’ (CA) (**Table 1**). The selected mandarin ‘W. Murcott’ is always propagated by grafting in commercial production. Therefore, the use of grafted plants in this study reflects standard commercial practices and ensures relevance to filed conditions. The individuals were divided into 1) non-infested and 2) infested plants and cultivated separately, under semi-field conditions, in two anti-aphid screened greenhouses (4 m × 8 m × 3.5 m) to avoid plant-to-plant communication. The plants were grown in pots (20 L) filled with a substrate mix consisting of peat: organic soil: perlite (2: 2: 1) and watered three times per week. The soil was provided with N:P:K in solution [Ultrasol^®^ Multipurpose 18-18-18, Soquimich, Chile] once a week. Up to the beginning of experiments, macro- and micronutrients were also supplied by foliar applications of Basfoliar SP 25-10-17 [COMPO EXPERT, Chile]. The meteorological data were obtained from a local weather station (**Supplementary Figure S1**).

**Table 1:** Main traits of ‘Macrophylla’ (*Citrus macrophylla* Wester); ‘C35’ [*C. sinensis* × *P. trifoliata* (South African)]; ‘Citrumelo’ (*Citrus paradisi* Macf. ‘Duncan’ grapefruit × *P. trifoliata*), and ‘Carrizo citrange’ (*Citrus sinensis* (L.) Osbeck × *Poncirus trifoliata* (L.) Raf.) rootstocks (Agusti et al., 2020).

Factors and characteristics	Rootstock			
	Macrophylla	C35	Citrumelo	Carrizo citrange
Biotic stress (BS)				
Phytophtora	+++	+++	+++	+++
Tristeza	+	+++	+++	+++
Citrus nematode	+	+++	+++	++
Exocortis	+++	+	+++	+
Xyloporosis	+	+++	+++	+
Psorosis	(-)	+++	+++	++
Abiotic stress (AS)				
Drought	+++	(-)	+++	+
Salt	+++	(-)	+++	+
Alkalinity	+++	++	+	+
Cold hardiness	+	+++	++	+++
Horticultural traits (Ht)				
Tree vigor	+++	++	+++	+++
Tree size	++	+	++	++
Fruit size	+++	++	+++	+++

+++ High resistant or high tolerance (*BS* & *AS*)/*Ht*: Large.

++ Intermediate tolerance (*BS* & *AS*)/*Ht*: Intermediate.

+ Low tolerance (*BS* & *AS*)/*Ht*: Small.

(-) No information.

### 2.2 Stock of *Panonychus citri* colonies

The citrus red mite *Panonychus citri* colonies were collected in citrus orchards at Pica Oasis, Chile (20°29’ S; 69°19’ W; 1,346 m.a.s.l.). It was reared on grapefruit (*Citrus* × *paradisi* Macfad.) fruits, placed on discs of PVC (diam. = 13 cm, H = 8 cm). To prevent the escape of mites, a small layer of vaseline was applied to contact surface between the fruit surface and the PVC disc. All inoculated fruits were put in a growth chamber at 25°C, 50% RH, and 16/8 h light/dark photoperiod.

### 2.3 Mite treatment and sampling

Infestation with mites was carried out in a greenhouse following a 30-day acclimation period. One shoot from the central part of the plant with 10 - 14 fully expanded leaves of each W. Murcott scion/rootstock combination was selected for treatment: a) WM/MA; b) WM/C35; c) WM/CI; d) WM/CA. The plants were inoculated with 20 gravid females of *P. citri* using a plastic micropipette tip. The tip was carefully attached by a clip to the abaxial side of each leaf, allowing *P. citri* to establish itself over the leaves. After 24 h, the micropipette tips and the clips were removed. The number of *P. citri* females was verified daily using a 10× handheld magnifying glass. Non-infested shoots with similar features as described above were chosen as controls. A seven-day infestation period was selected to ensure a robust and consistent physiological and molecular response to the imposed mite density of 20 adult females per leaf. This timeframe was chosen to precede the hatching of eggs, which typically occurs after approximately seven days and leads to a rapid and uneven increase in the *Panonychus citri* population. Extending the infestation period beyond this point could introduce uncontrolled variability due to asynchronous population growth and differing plant stress levels, thereby compromising the reproducibility of the observed responses.

At the end of the seventh day, the number of eggs per leaf from each scion/rootstock combination was assessed by counting under a stereoscope. Net assimilation and other photosynthesis-related parameters were evaluated using an infra-red gas analyzer (IRGA). Several leaves from control and treated plants were pooled in three different biological replicates. They were frozen in liquid nitrogen, freeze-dried and then stored until use. For RNA extraction, fresh leaf samples were collected in liquid nitrogen, and then stored at -80°C until analysis.

### 2.4 Stress-related biological markers

#### 2.4.1 Malondialdehyde content (MDA)

MDA content was determined according to Heath and Packer (1968) and Alfaro-Quezada et al. (2023). 

Approximately 0.25 g dry weight (DW) of leaf tissue was homogenized with 5 mL of a 5% trichloroacetic acid (TCA) solution and 1.25% glycerol. After centrifugation at 6,700 *g* for 10 min at 4°C and filtration through Whatman N° 1 filter paper, the supernatant was mixed with 2 mL of 0.67% thiobarbituric acid (TBA). The mixture was incubated for 30 min at 100°C, ice-cooled for 5 min, and centrifuged at 6,700 *g* for 1 min at 4°C. The absorbance was measured at 532 nm by UV-Vis spectrophotometry (BioTeK Instruments).

#### 2.4.2 Proline content

Proline content was determined in 0.5 g of leaf tissue by ninhydrin reaction (Bates et al., 1973). 

A standard curve using L-proline was made and absorbance was read at 520 nm by UV-Vis spectrophotometry.

#### 2.4.3 Salicilyc acid (SA) content

SA content was determined by a colorimetric reaction according to Warrier et al. (2013) with some modifications. 

About 0.05 g DW of leaf tissue was powdered and added with 1 mL of double distilled water, vortexed and placed in a dry bath at 60°C for 10 min. After centrifuging at 10,000 g for 10 min, an aliquot of 6.6 μL of supernatant was combined with 193.4 μL of fresh ferric chloride (FeCl). A SA (M.W. 138.12 g mol^-1^) standard was used for the calibration curve. The absorbance was read at 540 nm by UV-Vis spectrophotometry.

### 2.5 Gas exchange rates

The net assimilation rate of CO_2_ (*A*), stomatal conductance (*gs*), and transpiration (*E*) were measured using a portable apparatus (IRGA, LI-6800^®^, LI-COR Inc, Lincoln, Nebraska, USA). Fully expanded leaves from the central part of young trees were carefully extended and placed in the gas exchange chamber. The chamber was set to maintain a constant photosynthetically active radiation (PAR) of 1,200 µmol m^-2^s^-1^, and the carbon dioxide concentration (CO_2_) was held at 420 µmol mol^-1^ using the instrument’s internal CO_2_ injection system. The measurements were conducted at midday, between 11:30 a.m. and 12:30 p.m., on sunny days in February 2022, in three replicates per scion/rootstock combination and treatment.

### 2.6 Photosynthetic pigment, total sugar, and protein contents

#### 2.6.1 Photosynthetic pigment

About 0.5 g DW of leaf tissue were used for measuring chlorophylls and carotenoids (Lichtenthaler, 1987). 

Pigments were extracted in 10 mL of 80% (v/v) acetone and centrifuged for 10 min at 4,500 *g*. The absorbance was measured in the supernatant at 663, 646, and 470 nm by UV-Vis spectrophotometry.

#### 2.6.2 Total sugar content

Total sugar content was determined according to Dubois et al. (1956). 

Total sugars were extracted from 0.1 g DW using 5 mL of distilled water and shaken for 60 min. Then, they were centrifuged at 4,500 *g* for 30 min at 12 ± 2°C. An aliquot of 30 µL of supernatant was added with 180 µL of distilled water, 200 µL of phenol (80%), 1 mL of concentrated H_2_SO_4_ and cooled at RT in darkness. A standard curve with D-glucose was used, and the absorbance was read at 490 nm by UV-Vis spectrophotometry.

#### 2.6.3 Total protein content

Total proteins were extracted according to Nunes et al. (2015) with some modifications. 

About 1 g DW of leaf tissue was ground in buffer containing KH_2_PO_4_ 50 mM, pH 7.0, 2 mM EDTA, and 1% (w/v) PVP. The homogenate was then centrifuged at 10,000 *g* for 10 min at 4°C. The assay was performed using the Protein Assay Kit Pierce™ BCA (Thermo Scientific, USA) following the manufacturer’s instructions, with bovine serum albumin (BSA) as standard. The absorbance was read at 562 nm by UV-Vis spectrophotometry.

### 2.7 Total phenolic and flavonoid contents

Total phenolic and flavonoid contents were determined as described by Rao et al. (2019) with some modifications.

About 0.1 g of leaf tissue was homogenised with 5 mL of cooled 80% (v/v) methanol and shaken on an orbital shaker at 200 rpm for 2 h at RT. The homogenates were centrifuged at 2,500 g for 15 min. 

#### 2.7.1 Total phenolic content (TPC)

The TPC was determined in a 300-μl aliquot of the supernatant added with Folin reagent (Folin: distilled water 1: 10) and was incubated for 5 min at RT. Then, 2.25 mL of Na_2_CO_3_ solution (60 g L^-1^) was added and allowed to react in darkness for 2h at RT. The absorbance was measured at 725 nm using a UV-Vis spectrophotometer and the results are expressed in mg gallic acid equivalents (GAEs) per gram dry weight (mg GAEs g^-1^ DW).

#### 2.7.2 Total flavonoids content (TFC)

The TFC was determined in a 500 µL of methanolic extract combined with 2.25 mL of distilled water. 

An aliquot of 150 µL of 5% (w/v) NaNO_2_ in water solution was added and incubated for 6 min at RT. Then, 300 µL of 10% (w/v) of AlCl_3_ solution were added. After incubation at RT for 5 min, 1 mL of 1 M NaOH was added and vortexed for 30 s. The absorbance was measured by a UV-Vis spectrophotometer at 510 nm. The results are expressed as mg rutin equivalents (REs) per gram dry weight (mg REs g^-1^ DW).

### 2.8 VOCs collection and chemical analysis

**Figure 1:**
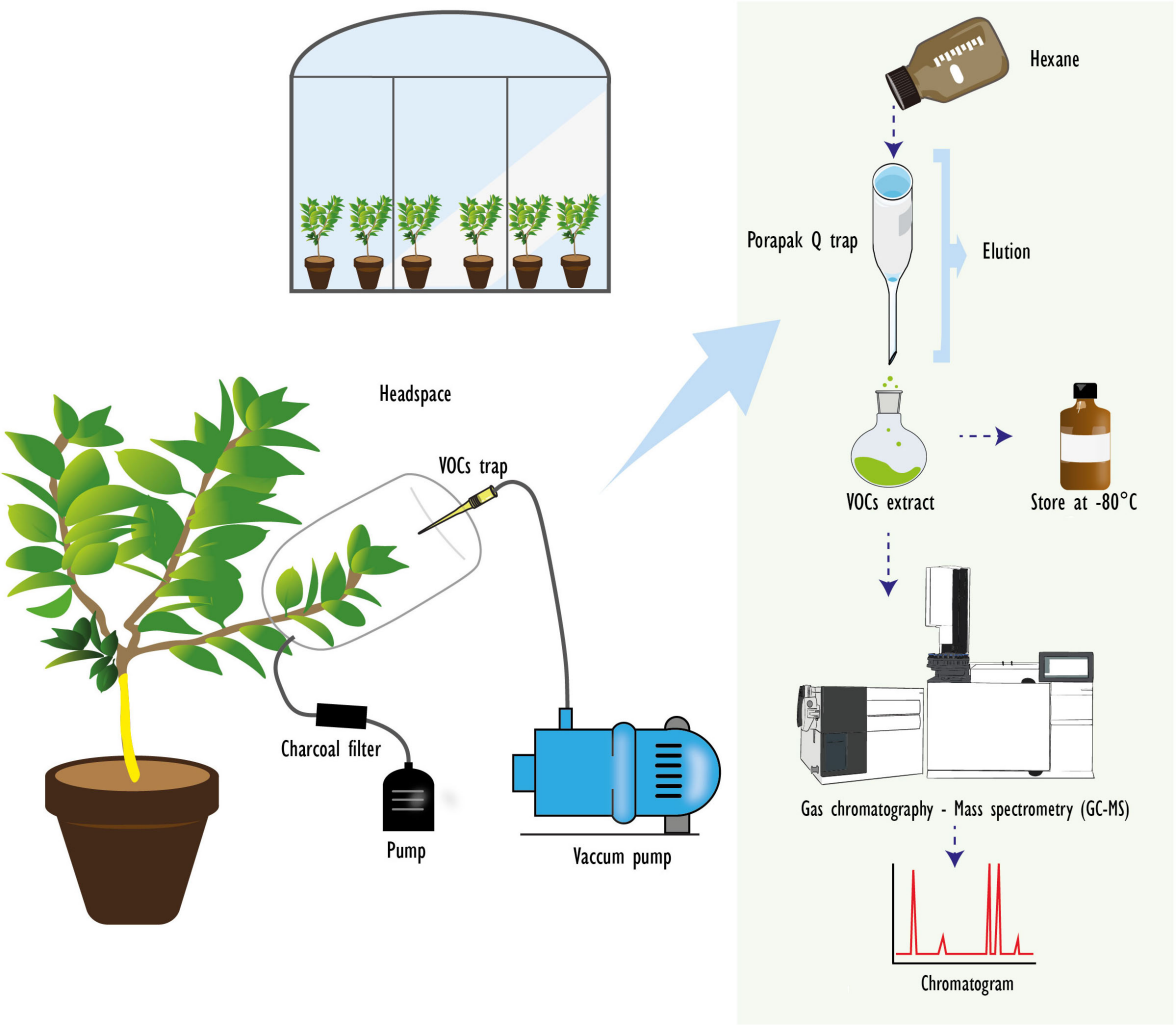
Dynamic headspace collection from citrus shoots under semi-field conditions. An air flow is passed through an activated charcoal filter and pumped into the bag. Simultaneously, the volatile organic compounds (VOCs) released from citrus shoots were extracted by a vacuum pump (BOECO, Hamburg, Germany), passing through glass traps filled with 100 mg of Porapak Q(™) (80-100 mesh, Waters Associates, Milford, Massachusetts, USA). The VOCs were eluted and stored in a laboratory ultrafreezer for chemical analyses and identification.

Volatile organic compounds (VOCs) were collected during the summer of 2022 using a dynamic headspace technique, as described by Rioja et al. (2016). Briefly, a shoot with 10–14 leaves was selected and enclosed in a 1-L oven bag (food-grade) while still attached to the plant. Filtered air (charcoal, 8–20 mesh, Sigma-Aldrich, St. Louis, Missouri, USA) was delivered into the bag at 1000 mL min⁻¹, and pulled it out at 900 mL min⁻¹ using a vacuum pump (BOECO, Hamburg, Germany) through a glass column containing 100 mg of Porapak Q adsorbent (80–100 mesh, Waters Associates, Milford, Massachusetts, USA) for 24 h (**Figure 1**). After sampling, each column was eluted with 1 mL of chromatographic-grade hexane (≥99%, Sigma-Aldrich) into a glass vial with PTFE-lined caps, and stored in amber vials at -80°C until chemical analysis. Porapak Q columns were cleaned and conditioned with 1 mL of redistilled diethyl ether (Merck, Darmstadt, Germany) under a nitrogen stream (70 mL min^-1^) at 150°C for 2 hours.

A 1-µL aliquot of the eluted VOCs was injected in splitless mode into a gas chromatograph coupled to a mass spectrometer (GC-MS; QP2010 Ultra, Shimadzu, Kyoto, Japan) equipped with an RTx5 capillary column (30 m, 0.25 mm internal diameter, 0.25 µm film thickness; Restek, Bellefonte, Pennsylvania, USA). The oven temperature program began at 40°C (held for 1 min), increased at 5°C min⁻¹ to 280°C, and was held for 5 min. Helium was used as carrier gas at a constant flow of 1 mL min⁻¹. Electron impact ionization was set at 70 eV, with a source temperature of 230°C, and mass spectra were acquired in the range of 50 to 500 m/z. VOC identification was carried out using LabSolutions GCMS software (v4.30, Shimadzu) and the NIST library (version 2.0). Although no retention indices or co-injection with authentic standards were conducted, compound identifications were based on high-quality spectral matches (≥90%) and are thus considered tentative unless otherwise specified. Quantification was conducted using the internal standard method, with tridecane (Sigma-Aldrich) as the analytical standard. Compound concentrations are expressed in µg mL^-1^.

### 2.9 Gene expression analysis by qRT-PCR

Total RNA was extracted from 100 mg of fresh leaf samples (Chang et al., 1993) collected three days after infestation. RNA yield and purity were checked by UV spectrophotometry, and RNA integrity was determined by electrophoresis. DNA was removed from 15 μg aliquots of total RNA using the TURBO DNA-free kit (Thermo, Applied Biosystems). The cDNA was synthesized from 6 μg of the DNaseI-treated RNA by means of the HighCapacity cDNA Kit (Thermo, Applied Biosystems), using random primers. Real-time qPCR was performed in a reaction mixture, final volume 25 µL, containing 100 *n*g of cDNA, 5 *p*mol of each primer, and 12.5 µL of the PowerUp SYBR Green PCR master mix (Thermo, Applied Biosystems), according to the manufacturer’s instructions. The oligonucleotides *CrEF1a* and *CrGAPDH*, annealing to the internal transcribed spacer of rRNA and encoding a member of the glyceraldehyde-3-phosphate dehydrogenase protein family, respectively, were used to amplify the internal standard with *Citrus* samples. The primer sequences used for the real-time qRT-PCR analysis are listed in Annexes (**Supplementary Table S1**; Agut et al., 2014; 2016). qPCRs were carried out using the QuantStudio(™) 3 Real-Time PCR System (ThermoFisher) following the kit instructions as follows: for 2 min at 50°C, 2 min at 95°C and then for 40 cycles of 95°C for 15 s and 60°C for 15 s, including the melt curve. The obtained Ct values were analyzed using the comparative threshold cycle or 2^-ΔΔCt^ method (Livak and Schmittgen, 2001). Transcript levels were normalized against *Elongation Factor 1-alpha* (*EF1a*), used as the internal reference gene due to its stable expression across all scion/rootstock combinations and under *Panonychus citri* infestation.

### 2.10 Experimental design and statistical analysis

A factorial design to determine the rootstock influence on physiological and biochemical traits in the commercial mandarin scions after seven days of continuous herbivory by *P. citri* was applied as follows: factor 1) four rootstock levels [(WM/MA), (WM/C35), (WM/CI), and (WM/CA)], and factor 2) infestation levels (non-infested or ‘control’, and infested plants). The physiological and biochemical parameters were evaluated using three biological replicates for each scion/rootstock combination with three technical replicates each. At least five biological replicates were collected *in vivo* to characterise the VOCs emitted by mandarin shoots. All data were transformed using natural logarithm (Ln) transformation [ln(x+1)] to meet normality requirements. To verify the effect of four levels of rootstocks on the physio- and biochemical traits of mandarin scion in two different infestation levels, a General Linear Model (GLM) was applied. This model is statistically equivalent to a two-way ANOVA. Where significant interaction effects were found, it was conducted an *post hoc* analysis using a one-way ANOVA followed by Tukey’s test (P < 0.05) to compare scion/rootstock combinations within infestation levels. Additionally, to compare non-infested *vs.* infested plants within each scion/rootstock combination were performed Student’s t-tests (P < 0.05). gene expression values are given as the mean of the normalized expression values of five technical replicates. Genes were considered up- or down- regulated when the fold change (FC) was ≥2 relative to the non-infested control. All statistical analyses were performed using software JASP (Version 0.19.3) (JASP Team, 2024).

## 3 Results

### 3.1 Physiological parameters linked to plant defence against mite attack

**Figure 2:**
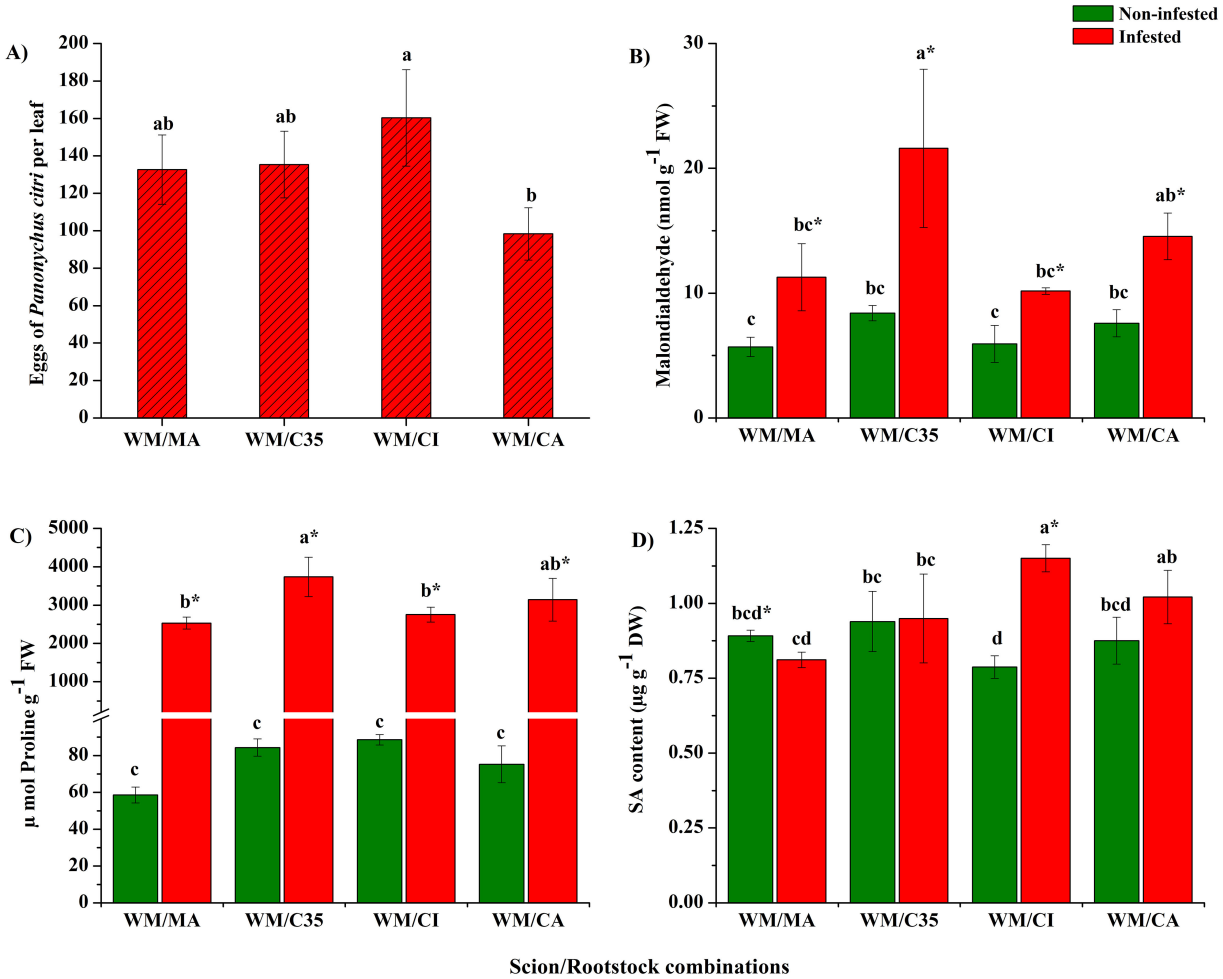
Oviposition preference expressed as number of eggs per leaf **(A)**, content of malondialdehyde (MDA; **B**), proline **(C)** and salicylic acid (SA; **D**) in citrus leaves infested with mites (*Panonychus citri*) on ‘W. Murcott’s mandarin grafted on different rootstocks: Macrophylla (MA), C35, Citrumelo (CI), and Carrizo (CA). Each bar indicates the mean of three biological replicates ± SD. Significant differences between scion/rootstock combinations in non-infested and infested plants were analyzed by ANOVA followed by Tukey’s *post-hoc* test (different letters = all against all; P < 0.05). The asterisk indicates statistically significant differences between control and infested leaves in the same scion/rootstock combination according to Student’s t-test (P < 0.05).

The oviposition preference by *P. citri*, calculated as the number of eggs per leaf, showed that all scion/rootstock combinations were significantly affected by the red mite attack (F = 5.13; P = 0.0287) (**Figure 2A**). After seven days of infestation, the females deposited 100 to 160 eggs per leaf. The scion/rootstock combination with lowest preference was CA, followed by MA and C35, while CI was the most affected by eggs deposition.

Lower MDA contents were recorded in non-infested mandarin leaves from all scion/rootstock combinations than infested ones (F = 2.60; P = 0.2269). However, after infestation, the MDA levels were significantly higher in the mandarin grafted on ‘C35’ (F = 14.35; *P =* 0.0323; **Figure 2B**); therefore, *P. citri* injury exacerbated rootstock influence on foliar MDA content in ‘W. Murcott’ scions (F = 3.40; *P =* 0.0434).

Proline contents of mandarin leaves were significantly enhanced by *P. citri* feeding in all scion/rootstock combinations (F = 574.27; P = 0.0001; **Figure 2C**). Furthermore, both factors. *i.e.*, rootstock and herbivory, affected the levels of this stress-protective metabolite (F = 8.39; P = 0.0014), with the ‘WM/C35’ combination yielding higher values than the others (**Figure 2C**).

As regards SA levels in mandarin leaves, results showed significant variations induced by rootstocks (F = 5.16; P = 0.0041; **Figure 2D**). Different from non-infested plants, SA contents increased significantly in infested mandarin grafted on ‘Citrumelo’ rootstock (F = 22.84; P = 0.0001). Thus, herbivory and rootstock interactions influenced foliar contents of SA (F = 17.58; P = 0.0001).

### 3.2 Rootstock influence on photosynthetic traits and pigments from ‘W. Murcott’ mandarin under mite attack

The net assimilation rate of CO_2_ (*A*) ranged from 9.356 to 11.518 µmol CO_2_ m^-2^ s^-1^ in control plants with the ‘WM/CI’ combination displaying the highest values. Seven days after infestation by the citrus red mite, *A* varied from 7.960 to 9.367 CO_2_ m^-2^ s^-1^ (F = 2.43; P = 0.0667; **Table 2**). The transpiration rate (*E*) of ‘W. Murcott’ mandarin leaves did not exhibit significant differences among the four scion/rootstock combinations and two *P. citri* infestation levels (F = 2.22; P = 0.0888; **Table 2**). By contrast, stomatal conductance (*gs*) varied depending on the scion/rootstock combination (F = 13.50; P = 0.0001), whereas herbivory had no effect on this parameter (F = 7.39; P = 0.0727).

**Table 2:** Physiological parameters and photosynthetic pigments of ‘W. Murcott’ mandarin leaves grafted onto ‘Macrophylla’ (*Citrus macrophylla* Wester), ‘C35’ [*C. sinensis* × *P. trifoliata* (South African)] ‘Citrumelo’ (*Citrus paradisi* Macf. ‘Duncan’ grapefruit × *P. trifoliata*), and ‘Carrizo citrange’ (*Citrus sinensis* (L.) Osbeck × *Poncirus trifoliata* (L.) Raf.) rootstocks under *Panonychus citri* attack under semi-field conditions.

Physiological parameters	Scion/rootstock combination							
	WM/MA		WM/C35		WM/CI		WM/CA	
	Control	Infested	Control	Infested	Control	Infested	Control	Infested
*A* (µmol m^-2^s^-1^)	10.52 ± 1.27 a	7.96 ± 1.33 a	10.79 ± 1.05 a	8.56 ± 1.48 a	11.52 ± 1.29 a	9.37 ± 1.93 a	9.36 ± 1.14 a	8.04 ± 1.69 a
*gs* (mol m^-2^s^-1^)	0.12 ± 0.01 bc	0.12 ± 0.02 c	0.17 ± 0.02 ab	0.15 ± 0.01 abc	0.18 ± 0.03 a	0.14 ± 0.02 abc	0.11 ± 0.02 c	0.11 ± 0.09 c
*E* (mol m^-2^s^-1^)	3.49 ± 0.33 a	3.32 ± 0.46 a	4.19 ± 1.02 a	3.42 ± 0.85 a	4.57 ± 0.57 a	3.68 ± 0.81 a	3.10 ± 0.34 a	2.87 ± 0.48 a
Leaf pigments								
*Chl a* (mg L^-1^)	18.90 ± 4.13 a	6.42 ± 1.99 b	24.76 ± 2.87 a	4.00 ± 0.95 b	22.96 ± 4.05 a	4.64 ± 0.84 b	21.35 ± 6.16 a	4.98 ± 0.93 b
*Chl b* (mg L^-1^)	8.23 ± 2.13 a	1.53 ± 0.33 b	12.91 ± 2.74 a	1.09 ± 0.34 b	11.28 ± 3.11 a	1.27 ± 0.23 b	10.47 ± 3.46 a	1.72 ± 0.38 b
*Chl a+b* (mg L^-1^)	27.15 ± 6.26 a	6.97 ± 0.99 b	37.69 ± 5.61 a	5.27 ± 1.29 b	34.26 ± 7.15 a	6.10 ± 1.07 b	31.84 ± 9.61 a	6.98 ± 0.98 b
*TC* (mg L^-1^)	4.43 ± 0.75 a	1.98 ± 0.45 b	5.92 ± 0.61 a	1.17 ± 0.25 b	5.33 ± 0.99 a	1.34 ± 0.17 b	4.50 ± 1.06 a	1.38 ± 0.34 b

The photosynthetically active radiation (PAR) was fixed at 1,200 µmol m^-2^ s^-1^, and the CO_2_ concentration was maintained at 420 µmol mol^-1^ using the equipment’s CO_2_ injection system. Values are expressed as means ± SD. *A*, net assimilation rate; *gs*, stomatal conductance; *E*, transpiration rate; *Chl* chlorophyll; *TC* total carotenoids. Significant differences between scion/rootstock combinations in non-infested (control) and infested plants were analyzed by ANOVA followed by Tukey’s post-hoc test (different letters within the same row = all against all; P < 0.05).

Rootstocks did not exert a significant influence on the concentrations of chlorophyll *a* and *b*, as well as carotenoids (chlorophyll *a*, F = 0.31; P = 0.8149; chlorophyll *b*, F = 1.09, P = 0.3828; chlorophyll *a+b*, F = 0.76, P = 0.5343; carotenoids, F = 0.89, P = 0.4658; **Table 2**). After seven days of herbivory, however, pigment concentrations for ‘W. Murcott’ tended to decrease significantly on all rootstocks (chlorophyll *a*, F = 160.99, P = 0.0001; chlorophyll *b*, F = 122.27, P = 0.0001; chlorophyll *a+b*, F = 152.96, P = 0.0001; carotenoids, F = 178.26, P = 0.0001).

### 3.3 Rootstock influence on biochemical and metabolic traits from ‘W. Murcott’ under herbivory

**Figure 3:**
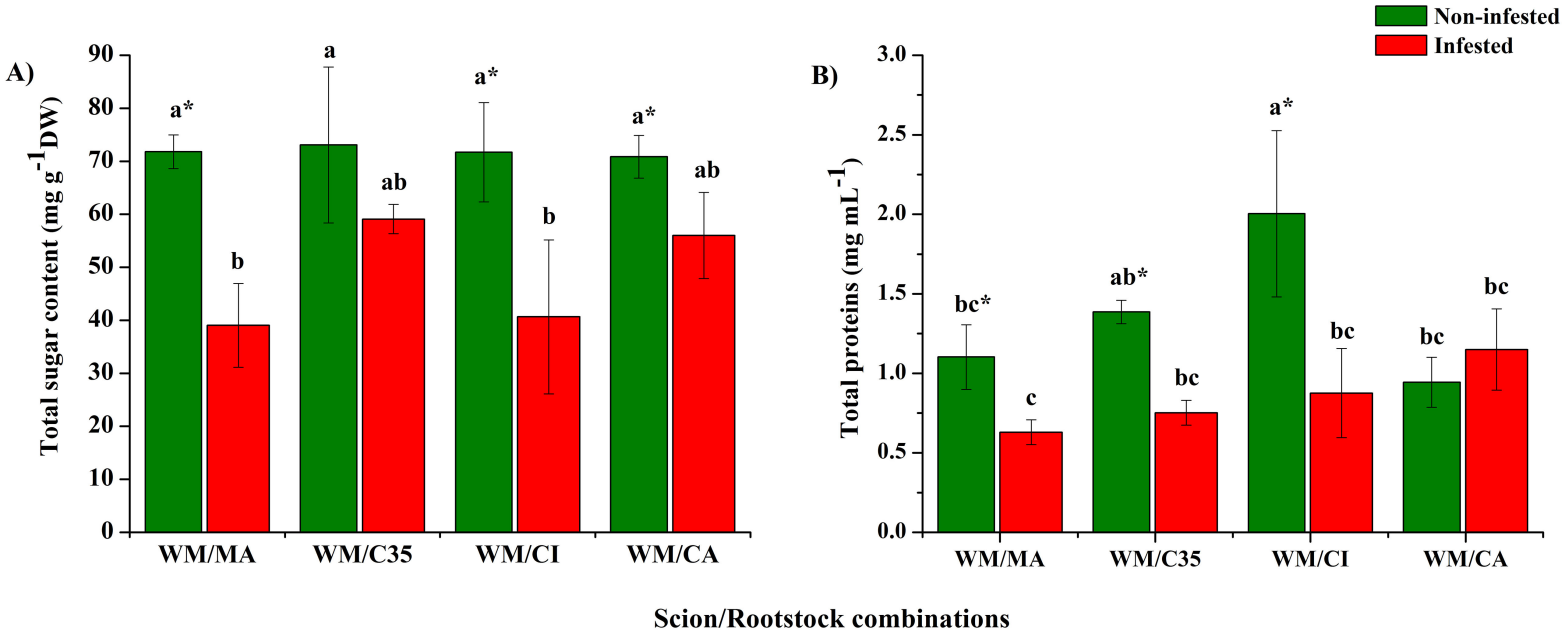
Total sugar **(A)** and soluble protein **(B)** contents in leaves of ‘W. Murcott’ mandarin scions grafted on Macrophylla (MA), C35, Citrumelo (CI), or Carrizo (CA) rootstocks and infested or not with mites *(Panonychus citri)* on Values are the means of five biological replicates ± SD. Significant differences between scion/rootstock combinations in control and non-infested plants were analyzed by ANOVA followed by Tukey’s *post-hoc* test (different letters = all against all; *P* < 0.05). Asterisks indicate statistically significant differences between control and infested leaves in the same scion/rootstock combination according to Student’s *t*-test (*P* < 0.05).

Citrus rootstocks did not affect soluble sugar levels (F = 1.09; P = 0.4727) in mandarin scions. However, these decreased significantly after *P. citri* injury when grafted on ‘MA’ and ‘CI’, going below constitutive levels of leaf sugar content (F = 20.91; P = 0.0196) (**Figure 3A**).

Soluble protein levels were higher in non-infested mandarin plants grafted on ‘Citrumelo’ than on other rootstocks (F = 9.09; P = 0.0001) (**Figure 3B**). Thus, results indicate that rootstocks influence leaf protein contents (F = 5.85; P = 0.0068). Moreover, after *P. citri* infestation, soluble proteins diminished in mandarin leaves in three of the four combinations compared to non-infested plants, except on the ‘CA’ rootstock (F = 27.17; P = 0.0001; **Figure 3B**), when it increased slightly.

**Figure 4:**
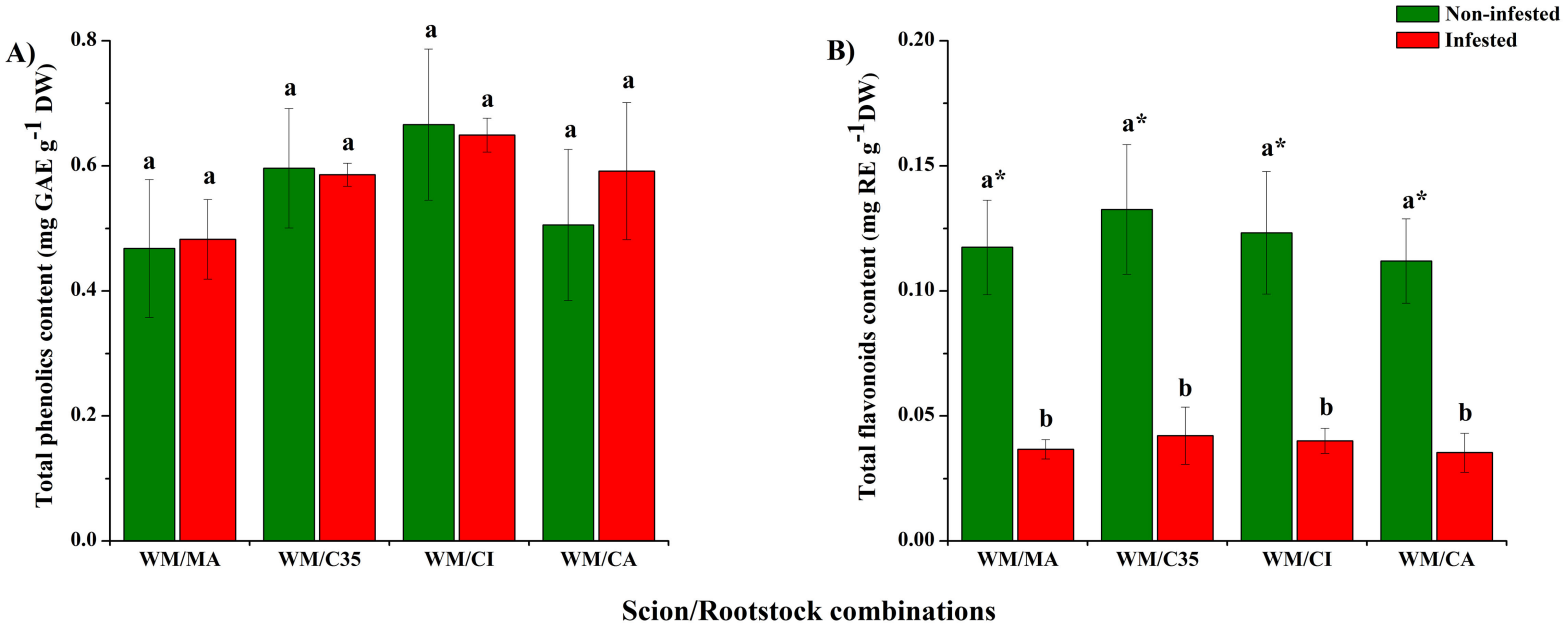
Total content of phenolic compounds **(A)** and flavonoids **(B)** in leaves of ‘W. Murcott’s mandarin grafted on Macrophylla (MA), C35, Citrumelo (CI), or Carrizo (CA). Rootstocks and infested or not with mites (*Panonychus citri*).: Values are the means of three biological replicates ± SD. Significant differences between scion/rootstock combinations in control and non-infested plants were analyzed by ANOVA followed by Tukey’s *post-hoc* (different letters = all against all; *P* < 0.05). Asterisks indicate statistically significant differences between control and infested leaves in the same scion/rootstock combination according to Student’s *t*-test (*P* < 0.05).

Rootstocks did not significantly affect TPC (F = 4.14; P = 0.2037; **Figure 4A**), which ranged between 0.46 ± 0.11 and 0.67 ± 0.12 mg GAEs g^-1^ DW, nor TFC (F = 0.77; P = 0.5268; **Figure 4B**) in mandarin leaves. On all rootstocks, TFC, that ranged from 0.035 ± 0.007 to 0.042 ± 0.011 mg REs g^-1^ DW, diminished significantly (at least three-fold) after *P. citri* infestation (F = 155.82; P = 0.0001; **Figure 4B**) compared to non-infested plants.

### 3.4 Scion/rootstock interaction on VOCs emitted by ‘W. Murcott’ leaves infested with *P. citri*

As shown in **Table 3**, chemical profiles showed slight differences between ‘W. Murcott’/rootstock combinations, mainly with respect to undetected compounds, whereas 2,3,3-trimethylhexane, and 2,4-dimethylhept-1-ene were only registered from scions grafted on ‘MA’. hexan-3-ol (3-hexanol), 2,2,4-trimethyldecane and 3-ethylbenzaldehyde were identified only in two or three scion/rootstock combinations. After seven days of infestation, (1R)-2-methyl-5-propan-2- ylbicyclo [3.1.0] hex-2-ene (α-thujene), [(Z)-hex-3-enyl] acetate (cis-3-Hexenyl acetate), methyl 2-hydroxybenzoate (methyl salicylate, MeSA), (3E, 6E) - 3, 7, 11 -trimethyldodeca-1,3,6,10-tetraene (α-farnesene),(3E)-3,7-dimethylocta-1,3,6-triene(β- ocimene), 3,7-dimethylocta-1,6-dien-3-ol (linalool), and (6R)-3-methylidene-6-propan-2-ylcyclohexene (β-phellandrene) were detected only from infested scions (**Table 3**). Moreover, significant variations were caused by rootstocks after herbivory (**Supplementary Figure S2**); thus, HIPVs released from ‘W. Murcott’ scions, such as (1S,5S)-6,6-dimethyl-2-methylidenebicyclo[3.1.1]heptane (β-pinene), cis-3-Hexenyl acetate, and linalool, varied significantly between scion/rootstock combinations (**Table 3**). The amounts of MeSA and (4R)-1-methyl-4-prop-1-en-2-ylcyclohexene (D-limonene) released from infested shoots did not show significant differences in any of the scion/rootstock combinations (**Table 3**). Although identification was not confirmed via retention indices or co-injection with authentic standards, the observed differences in compound abundance support biologically meaningful interpretations and provide a basis for selecting candidate VOCs for functional assays.

**Table 3:** Concentrations of volatile organic compounds emitted from (*Citrus reticulata* Blanco) ‘W. Murcott’ scion grafted onto ‘Macrophylla’ (*Citrus macrophylla* Wester), ‘C35’ (*C. sinensis* × *P. trifoliata* (South African)) ‘Citrumelo’ (*Citrus paradisi* Macf. ‘Duncan’ grapefruit × *P. trifoliata*), and ‘Carrizo citrange’ (*Citrus sinensis* (L.) Osbeck × *Poncirus trifoliata* (L.) Raf.) rootstocks. Values represent mean concentration (n = 6) ± standard error (µg mL^-1^) of volatile organic compounds (VOCs) identified based on ≥90% spectral match with the NIST library (v2.0).

Compound*	Scion/Rootstock							
	W. Murcott/MA		W. Murcott/C35		W. Murcott/CI		W. Murcott/CA	
	Control	Infested	Control	Infested	Control	Infested	Control	Infested
Alcohol								
hexan-3-ol (3-hexanol)	–	–	1.20 ± 0.04 B	–	1.69 ± 0.34 A	–	–	–
Alkanes								
2,3,3-trimethylhexane	4.40 ± 1.17a	9.01 ± 2.33 Aa	–	14.32 ± 1.26 A	–	8.38 ± 2.00 A	–	10.46 ± 1.71 A
2,4-dimethylhept-1-ene	5.02 ± 1.51a	7.13 ± 1.36 Aa	–	–	–	–	–	7.92 ± 1.13 A
6-ethyl-2-methyldecane	–	8.46 ± 1.87 AB	13.64 ± 0.77 Aa	3.83 ± 2.06 Bb	21.76 ± 2.89 Aa	12.40 ± 3.08 Aa	15.80 ± 3.04 Aa	1.28 ± 0.27 Bb
2,2,4-trimethyldecane	27.1 ± 6.16 A	–	25.82 ± 5.15 A	–	–	–	26.45 ± 8.39 A	–
3-methylheptadecane	–	26.57 ± 4.12	–	–	–	–	–	–
2,2-dimethylicosane	29.85 ± 10.09 Aa	44.12 ± 7.69 Aa	41.23 ± 10.56 Aa	37.55 ± 2.96 ABa	35.97 ± 13.06 Aa	22.54 ± 4.65 Ba	37.42 ± 10.71 Aa	37.15 ± 3.69 ABa
pentadecane (n-Pentadecane)	155.22 ± 36.72 Aa	54.58 ± 10.32 Aa	177.62 ± 32.01 Aa	27.68 ± 1.99 Ab	169.15 ± 42.27 Aa	51.60 ± 25.12 Ab	100.29 ± 29.74 Aa	29.22 ± 3.21 Aa
tetradecane (n-Tetradecane)	21.64 ± 5.09 Ab	205.35 ± 45.79 Ba	23.48 ± 4.58 Ab	220.13 ± 11.94 ABa	98.71 ± 76.16 Aa	604.71 ± 191.82 Aa	18.80 ± 5.72 Ab	149.64 ± 15.80 Ba
hexadecane (n-Hexadecane)	–	112.01 ± 34.82 A	–	56.10 ± 4.43 A	–	113.54 ± 25.71 A	–	127.20 ± 11.44 A
Benzaldehydes								
4-ethylbenzaldehyde (4-ethyl benzaldehyde)	29.22 ± 5.99 Aa	18.27 ± 4.05 Ba	18.93 ± 2.50 Ab	28.49 ± 2.13 ABa	–	34.34 5.18 AB	32.28 ± 8.45 Aa	36.06 ± 4.77 Aa
3-ethylbenzaldehyde (3-Ethylbenzaldehyde)	50.26 ± 9.45 A	–	36.99 ± 3.94 A	–	–	–	46.59 ± 13.37 A	–
Esters								
[(Z)-hex-3-enyl] acetate (cis-3-Hexenyl acetate)	–	71.84 ± 9.61 A	–	17.07 ± 1.61 B	–	74.48 ± 11.43 A	–	6.38 ± 1.61 C
methyl 2-hydroxybenzoate (methyl salicylate)	–	36.16 ± 8.69 A	–	44.58 ± 2.63 A	–	41.89 ± 6.52 A	–	31.47 ± 3.07 A
Hydrocarbons								
1,2,3,6-tetramethylbicyclo[2.2.2]octa-2,5-diene	–	47.90 ± 5.89 A	–	37.01 ± 2.56 A	–	124.83 ± 41.81 A	–	41.50 ± 1.31 A
Ketones								
Hexan-3-one (3-Hexanone)	2.84 ± 0.74 Aa	1.79 ± 0.40 ABa	2.39 ± 0.48 Aa	2.70 ± 0.26 Aa	2.93 ± 0.45 Aa	1.10 ± 0.16 Bb	2.20 ± 0.85 Aa	2.16 ± 0.12 Aa
Hexan-2-one (2-Hexanone)	4.51 ± 1.04 Aa	2.29 ± 0.50 ABa	–	3.54 ± 0.18 A	3.96 ± 0.38 Aa	1.34 ± 0.28 Bb	2.43 ± 1.17 Aa	2.63 ± 0.29 ABa
1-(4-ethylphenyl)ethan-1-one	782.05 ± 221.14 Aa	577.13 ± 102.43 ABa	415.58 ± 57.94 Ab	731.49 ± 53.35 ABa	–	393.71 ± 209.15 B	625.37 ± 187.45 Aa	977.95 ± 64.68 Aa
Monoterpenes								
(1R)-2-methyl-5-propan-2-ylbicyclo[3.1.0]hex-2-ene (α-thujene)	–	3.21 ± 1.38	–	–	–	7.43 ± 1.21	–	–
(1S,5S)-6,6-dimethyl-2-methylidenebicyclo[3.1.1]heptane (β-pinene)	–	4.31 ± 1.24 B	–	3.62 ± 1.28 B	5.49 ± 0.76 b	62.83 ± 8.81 Aa	–	3.56 ± 0.60 B
(3E)-3,7-dimethylocta-1,3,6-triene (β-ocimene)	–	–	–	19.82 ± 5.38 A	–	27.37 ± 2.56 A	–	20.19 ± 1.54 A
(4R)-1-methyl-4-prop-1-en-2-ylcyclohexene (D-limonene)	19.41 ± 4.82 Aa	24.36 ± 7.64 Aa	12.05 ± 2.02 Ab	47.10 ± 8.61 Aa	19.73 ± 4.93 Aa	21.90 ± 1.28 Aa	10.02 ± 2.14 Ab	45.29 ± 1.48 Aa
(6R)-3-methylidene-6-propan-2-ylcyclohexene (β-phellandrene)	–	–	–	–	–	7.84 ± 1.54	–	–
Sesquiterpenes								
(3E,6E)-3,7,11-trimethyldodeca-1,3,6,10-tetraene (α-farnesene)	–	35.19 ± 3.17 A	–	27.71 ± 1.63 A	–	38.65 ± 8.39 A	–	41.02 ± 2.50 A
3,7-dimethylocta-1,6-dien-3-ol (linalool)	–	–	–	21.99 ± 3.06 B	–	39.32 ± 7.80 AB	–	45.27 ± 5.22 A

A General Linear Model (GLM) was used to assess the effects of rootstock, infestation, and their interaction. Different uppercase letters within a row and within the same condition (control or infested) indicate significant differences among scion/rootstocks (P < 0.05, Tukey’s HSD). Different lowercase letters within the same row and same scion/rootstock combination indicate significant differences between control and infested plants (P < 0.05, Student’s t-test). “–” indicates that the compound was not detected. *IUPAC name (common name).

### 3.5 Gene expression of infested scions during *P. citri* attack

**Figure 5:**
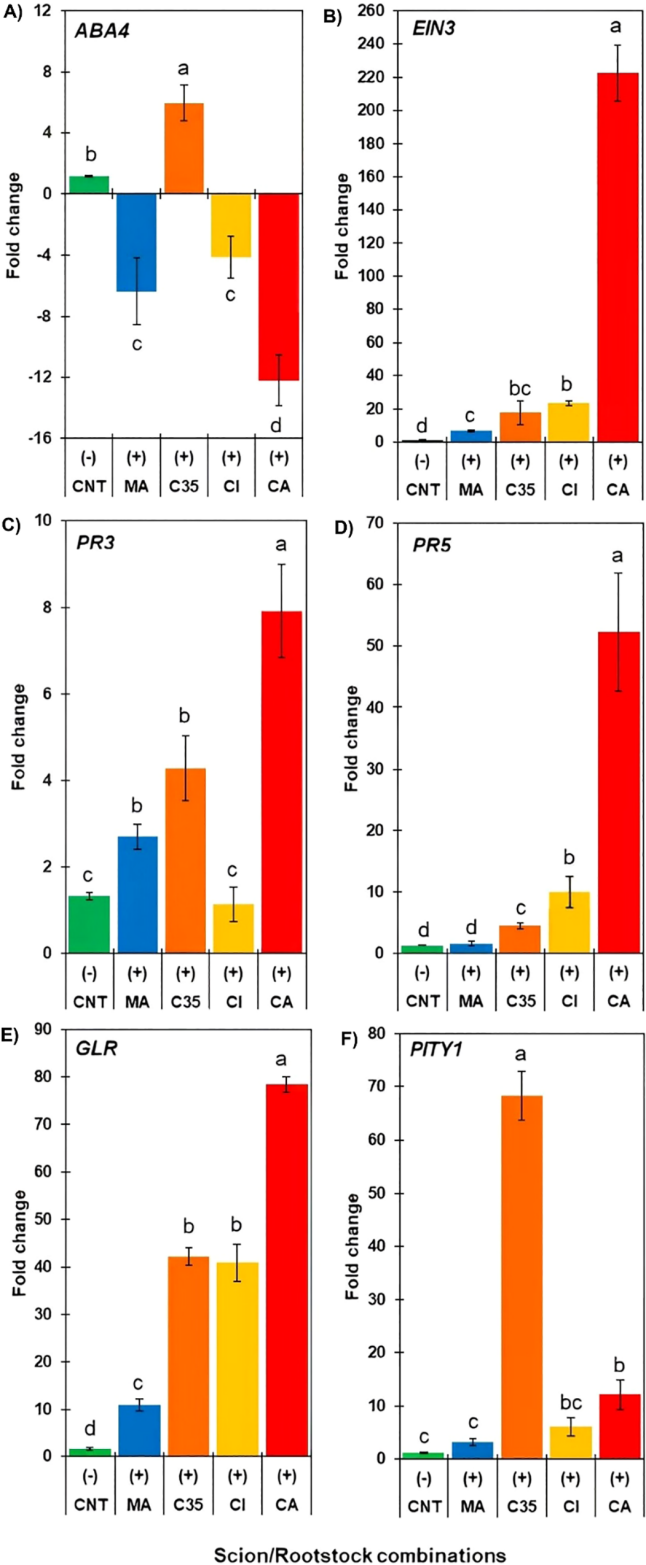
qRT-PCR analysis (FC, fold change) of genes related to phytohormone biosynthesis [**(A)** abscisic acid **(B)**] and ethylene) and defence **(C–F)** in mandarin scions grafted on different *Citrus* rootstocks (MA, C35, CI, and CA) seven days after being infested with red mite. Expression values are means of five technical replicates, normalized against *EF1a* as a reference gene. Gene expression was analyzed relative to a pooled non-infested control (CNT), composed of all non-infested scion/rootstock combinations, and calculated separately for each gene. Genes were considered up- or down-regulated when fold change (FC) relative to the non-infested control on the same rootstock (CNT) was ≥ 2. Different letters indicate statistically significant differences among combinations according to Tukey’s test (P < 0.05). Error bars represent standard error of the mean.

This section presents gene expression data from infested scions. The control (fold change = 1) corresponds to a pooled baseline composed of all non-infested scion/rootstock combinations, calculated separately for each transcript. Specifically, CNT represents the average Ct value of non-infested controls across all rootstocks (**Supplementary Figure S3**) shows the Ct values of non-infested controls for each rootstock). This pooled control was used to normalize gene expression levels, allowing consistent comparison of infestation-induced responses among genotypes. Of all the genes explored, *EIN3*, *PR3*, and *GLR* did not show detectable and stable levels of transcripts through replicates in non-infested control ‘W. Murcott’ scions. Transcript accumulation of *ABA4* (**Figure 5A**) was significantly down-regulated in scions grafted onto ‘CI’, ‘CA’, and ‘MA’ rootstocks, up to 12-fold in the case of ‘Carrizo citrange’. Conversely, ‘C35’ rootstock significantly increased *ABA4* transcript levels in response to insect attack.

*EIN3* was significantly up-regulated in mandarin scions grafted onto ‘CI’, ‘C35’, and ‘CA’ rootstocks, with the latter being upregulated over 200-fold compared to the control. It exhibits one of the highest expression levels among the genes evaluated in this study (**Figure 5B**).

The expression of *PR3* was significantly higher in the ‘W. M./CA’ combination, reaching up to 8-fold control levels; ‘WM/MA’ and ‘WM/C35’ combinations also showed a lower but significant increase, while on ‘Citrumelo’ rootstock, scions maintained similar transcript levels to the controls (**Figure 5C**). As for *PR5*, ‘WM/CA’, ‘WM/CI’, and ‘WM/C35’ showed a significant increase compared to the control, while ‘WM/MA’ remained unchanged in response to red mite herbivory. *PR3* was generally less expressed than *PR5*, the latter being up-regulated up to 50-fold control level ‘WM/CA’ (**Figure 5D**). The expression of *GLR* significantly increased for all rootstocks in response to red mite attacks, with scions grafted on ‘CA’ showing the most significant increase (80-fold over the control), followed by ‘CI’, ‘C35’, and ‘Macrophylla’ (10-fold control levels; **Figure 5E**). *PITY1* transcript accumulation significantly increased in mandarin grafted on ‘C35’ (up to 70-fold over the control), followed by ‘CA’ (12-fold), while ‘CI’ and ‘MA’ did not induce significant differences compared to the control (**Figure 5F**).

## 4 Discussion

### 4.1 Red mite attack activates stress responsive biological markers

The number of eggs of *P. citri* after seven days of infestation ranged from approximately 100 to 160 eggs per leaf and can be considered detrimental to the physio-biochemical functioning of W. Murcott, as in other mandarin cultivars (Agut et al., 2016).

MDA is widely used as a marker of membrane integrity and stress tolerance in plants (Morales and Munné-Bosch, 2019; Sheri et al., 2023), as it activates regulatory genes related to plant defence and development. After seven days of continuous *P. citri* infestation, all scion/rootstock combinations showed elevated MDA levels, with ‘WM/C35’ exhibiting the highest. Similar MDA increases have been reported in *T. urticae* - infested bean plants (Farouk and Osman, 2012), whereas in cucumber, MDA initially rose but later declined under sustained mite feeding (Shahtousi and Talaee, 2023), suggesting that lower levels of MDA may reflect reduced damage, greater antioxidant capacity, and increased tolerance to herbivores. Our findings support the idea that both ‘C35’ and ‘CA’ rootstock are less effective in limiting membrane damage, as reflected by higher MDA accumulation. This agreed with increased proline and *ABA4* expression in the ‘C35’ rootstock/scion combination, pointing to greater susceptibility to red mite attack.

The osmolyte proline has been extensively studied in grafted citrus under abiotic stressors such as salinity, drought, and heat (Shahid et al., 2019; Balfagón et al., 2022). Our results show that the different rootstocks did not significantly influence constitutive levels of this stress-protective compound. By contrast, insect attacks are known to induce osmolyte accumulation, including proline, as reported in plants infected by fungi, viruses, or infested by *T. urticae* (Qamar et al., 2015; Anzano et al., 2022; Farouk and Osman, 2012). Notably, proline levels decrease in mite-susceptible wild rice *(Oryza barthii*) leaves but increase in tolerant cultivars following *Schizotetranychus oryzae* attack (Acari: Tetranychidae; Buffon et al. (2021). Similarly, we observed a significant post-infestation increase in proline across all scion/rootstock combinations, with the highest levels in ‘C35’ rootstock. This suggest that while constitutive proline remained stable, its inductibility under mite stress may reflect an active, though not necessarily protective, response.

Our results show that basal SA levels in mandarin leaves were rootstock-dependent, with the lowest levels observed in the ‘CI’ combination. This agrees with Agut et al. (2016), who found significant differences in constitutive SA content in ‘Clemenules’ scions grafted onto different rootstocks. Beyond its developmental roles, SA is a key phytohormone in plant defence (Mishra et al., 2024). Upon *T. urticae* infestation, SA levels increased in ‘Clemenules’/’Cleopatra’ after three days, while ungrafted ‘Sour orange’ exhibited higher SA than ‘Cleopatra’ after seven days (Agut et al., 2014, 2016). Similarly, Leus et al. (2022) reported elevated SA in *R. simsii* cultivars infested by *P. latus*. Consistent with these findings, we observed significant SA accumulation in ‘W. Murcott’ grafted onto ‘CI’ and ‘CA’ rootstocks following seven days of *P. citri* infestation.

### 4.2 Scion/rootstock combinations modulates photo-assimilation and photosynthetic pigments under red mite attack

Yulianti and Agisimanto (2023) reported that grafting ‘Pontianak’ tangerine onto ‘Japansche citroen’ and ‘Citrumelo’ rootstocks significantly affected photosynthetic rate, whereas no differences were observed with ‘Montaji’ lemon. In our study, although *P. citri* infestation led to reductions in *A* and *E* in ‘W. Murcott’, the differences were not significant; however, *g_s_* was significantly influenced by rootstock. Similarly, Yulianti and Agisimanto (2023) observed no *gs* variation in their grafting experiments. Earlier, Hare and Youngman (1987) found no significant physiological changes in ‘Washington Navel’ oranges infested by *P. citri*, suggesting leaf tolerance. In contrast, *T. urticae* infestation significantly reduced photosynthesis in cotton (Reddall et al., 2007) and lowered *A*, *gs*, and *E* in *J. curcas* (Hsu et al., 2015). Overall, our data suggest that ‘W. Murcott’ grafted onto the tested rootstocks tolerates *P. citri* infestation for at least seven days without marked impairment of photosynthetic performance.

Photosynthetic pigments are known to be affected by biotic stresses (Li et al., 2024). In our study, rootstocks did not alter chlorophyll or carotenoid levels in mandarin leaves, but *P. citri* feeding significantly reduced both pigments, potentially contributing to the observed, albeit non-significant, decline in photosynthetic parameters. Similarly, Blasi et al. (2017) reported chlorophyll loss in *S. oryzae*-infested rice leaves. In contrast, *T. urticae* infestation increased carotenoid content in beans (Farouk and Osman, 2012), while *J. curcas* showed no pigment changes under similar infestation (Hsu et al., 2015).

### 4.3 Rootstocks influence primary and secondary metabolites in ‘W. Murcott’ leaves under mite attack

Our results show that citrus rootstocks did not significantly affect constitutive soluble sugar levels in ‘W. Murcott’ mandarin. However, following *P. citri* infestation, sugar content declined significantly, particularly in plants grafted onto ‘CI’ and ‘MA’. This aligns with studies reporting sugar level changes under herbivory: increases in *T. urticae*-infested beans (Farouk and Osman, 2012) and *T. evansi*-infested tomato (Ximénez-Embún et al., 2018), but decreases in *J. curcas* (Hsu et al., 2015). Hayat et al. (2022) also noted rootstock-driven sugar variability in mandarin leaves, with ‘Trifoliate Orange’ inducing the highest content. The observed sugar depletion in our study may reflect a resource reallocation strategy under biotic stress, where breakdown of reserves contributes to defence signaling (Van den Ende and El-Esawe, 2014). In particular, the ‘WM/CI’ combination appears especially reactive to mite attack, mirroring findings in sugarcane under aphid pressure (Koch et al., 2020).

Soluble proteins are crucial for plant growth and defence against biotic stress (Han et al., 2023). In our study, ‘W. Murcott’ grafted onto ‘CI’ exhibited significantly higher basal protein levels, indicating a rootstock effect. Similar influences have been reported in ‘Shatangju’ and ‘March Seedless’ grafted onto protein-promoting rootstocks like ‘Citrange’, ‘Flying Dragon’, and ‘Troyer citrange’ (Hayat et al., 2022; Sharma et al., 2015). Upon *P. citri* infestation, protein levels declined significantly across most combinations, except for ‘CA’, suggesting a rootstock-dependent response. Such reductions mirror those observed in *T. urticae*-infested *J. curcas* and *T. evansi*-infested tomato (Hsu et al., 2015; Ximénez-Embún et al., 2018). Mite-secreted effectors are known to manipulate plant proteomes, including components of the ubiquitin-proteasome system, autophagy, phytohormone signaling, and transcription regulation (Blaazer et al., 2018; Zhao and Wang, 2024). Likewise, *S. oryzae* infestation down-regulated defence- and metabolism-related proteins in rice (Blasi et al., 2017). These findings suggest that *P. citri* may similarly suppress host protein-based defences to enhance its fitness.

Rootstocks did not significantly affect constitutive total phenolic content (TPC) in ‘W. Murcott’, although higher levels were observed in scions grafted onto ‘C35’ and ‘CI’. Similarly, Legua et al. (2014) found increased TPC in ‘Clemenules’ grafted onto ‘Volkameriana’. After seven days of *P. citri* infestation, TPC remained unchanged in most combinations but showed an increasing trend in ‘WM/CA’. In contrast, *T. urticae* infestation led to significantly higher TPC in beans (Farouk and Osman, 2012), suggesting species-specific or stress duration-dependent phenolic responses.

As with TPC, rootstocks did not influence constitutive flavonoid content in ‘W. Murcott’ leaves. However, other studies have shown rootstock effects: higher flavonoid levels were reported in ‘Maltese half-blood’ orange grafted onto ‘Volkameriana’ (Zouaghi et al., 2018) and in ‘Newhall’/P. trifoliata compared to ‘Newhall’/C. junos (Li et al., 2023). Flavonoids contribute to plant defence through deterrent and antifungal properties and accumulate in response to bacterial infections (Anzano et al., 2022). In our study, total flavonoid content (TFC) significantly declined after seven days of *P. citri* infestation, suggesting mite-mediated suppression of defence pathways. This is consistent with findings in tomato, where *T. evansi* and *T. urticae* reduced flavonoid levels and suppressed associated signalling pathways (Knegt et al., 2020; Su et al., 2020). *P. citri* may act similarly, downregulating flavonoid-dependent defences in mandarin.

Citrus VOC emissions are influenced by rootstocks (Jones and Killiny, 2021; Guarino et al., 2022). For example, ‘Minneola’ grafted onto ‘MA’ releases β-phellandrene, caryophyllene, citronellol, and cis-p-mentha-2,8-dien-1-ol—compounds absent in lime leaf emissions (Rioja and Ceballos, 2024). Rootstock-dependent changes in VOC profiles have also been observed under *Citrus tristeza virus* infection (Guarino et al., 2022). In our study, ‘W. Murcott’ VOC profiles were only slightly affected by rootstock, with minor variations in alcohols, alkanes, and aromatic aldehydes across combinations, similar to findings by Jones and Killiny (2021). Herbivory can trigger the release of HIPVs as indirect defences. For instance, increased emissions of D-limonene, ocimene, and MeSA have been documented in citrus infested by *Aonidiella aurantii* (Alsabte et al., 2022), and higher levels of MeSA, azulene, and 2-ethylhexan-1-ol were detected in mite-infested ‘Minneola’ (Rioja and Ceballos, 2024). In avocado, *O. yothersi* induced exclusive emissions of β-ocimene, linalool, α-farnesene, and MeSA, which also act as repellents (Rioja et al., 2016, 2018). In our study, MeSA was consistently detected in all *P. citri*-infested scion/rootstock combinations, reinforcing its role as a key HIPV mediating tri-trophic interactions (Abdala-Roberts et al., 2019). Seemingly, the citrus rootstocks do not appear to affect the tritrophic interactions; therefore, studies on the behavioural responses in predators of *P. citri* are required. Present results show that the chemical profiles changed both quantitatively and qualitatively after *P. citri* infestation. High emissions of α-thujene, β-pinene, and β-phellandrene were registered only in the ‘WM/CI’ combination, indicating that citrus rootstocks markedly affect the indirect induced defences in mandarin scions.

### 4.4 Scion/rootstock combinations differentially affect phytohormone- and defence- related genes under mite attack

Gene expression is a sensitive indicator of plant responses of early molecular responses to stress (Wang et al., 2023). Phytohormones like ethylene (ET) and abscisic acid (ABA) modulate defence gene expression, often via antagonistic pathways (Li et al., 2019; Yu et al., 2021; Müller, 2021). *ABA4*, which encodes a membrane protein involved in neoxanthin synthesis and stress-induced ABA accumulation (North et al., 2007), was generally downregulated in our study, except in the ‘C35’ combination, where it was significantly upregulated. Elevated *ABA4* expression, along with high MDA levels, suggests greater membrane damage and stress in this rootstock under *P. citri* infestation, consistent with responses observed in *A. thaliana* (Barczak-Brzyżek et al., 2017; Rosa-Diaz et al., 2024), *Tamarix nilotica* (Younis, 2021), and ‘Cleopatra’ mandarin (Agut et al., 2014). Conversely, *EIN3*, an ET-responsive transcription factor, was strongly upregulated across all combinations, reaching a 220-fold increase in ‘WM/CA’. *EIN3* is a central regulator of ET signaling and downstream defence responses (Solano et al., 1998; Binder, 2020; Pérez-Hedo et al., 2024) and has been similarly induced in cassava infested by *T. urticae* (Yang et al., 2019). These findings highlight distinct hormonal response strategies to mite attack among rootstock combinations.

Pathogenesis-related (PR) proteins play key roles in plant defence by reinforcing cell structures and exerting enzymatic activity against pathogens (Van Loon et al., 2006; Han et al., 2023). Members of the PR family have specific functions: chitinases (PR-3) act via the JA pathway, while thaumatin/osmotin-like proteins (TLPs; PR-5) are SA-responsive. In our study, both *PR-3* and *PR-5* were strongly upregulated by *P. citri* infestation in ‘WM/CA’ and ‘WM/C35’ combinations, with *PR-5* reaching transcript levels ten times higher than *PR-3*. *PR-5* is known to be recruited by *PR-1* to enhance resistance through ROS-dependent amplification of immune responses (Han et al., 2023). The accumulation of *PR* gene transcripts is a hallmark of SA- and JA-mediated defence and is associated with the production of antimicrobial proteins such as glucanases (PR-2), chitinases (PR-3, PR-4), and TLPs (PR-5; Gkizi et al., 2016). In citrus infested by *Tetranychus* spp., *PR-5* expression increases early, while *PR-3* induction is delayed but sustained (Agut et al., 2014, 2015), consistent with our findings.

Some mite species can suppress defence-related gene expression. In tomato (*S. lycopersicum* var. Santa Clara I-5300), *T. evansi* suppressed *WIPI-II* and *PR-P6*, genes linked to JA and SA pathways, respectively, whereas *T. urticae* upregulated both (Sarmento et al., 2011). Similarly, in azalea, *P. latus* initially induced JA accumulation, but later significantly increased SA levels, suggesting suppression of JA-mediated defences to enable sustained infestation without compromising mite fitness (Leus et al., 2022).

The citrus *Protein Inhibitor Type 1* (*PI TYPE1*) gene is a known marker of arthropod-induced defence (Agut et al., 2014). In our study, *PITY1* was significantly upregulated across all scion/rootstock combinations, with the highest expression observed in ‘WM/C35’ (65-fold), followed by ‘CA’ (12-fold), and lower increases in ‘CI’ and ‘MA’ (7- and 3-fold, respectively). Similar strong induction of *PI* genes has been reported in tomato under *T. urticae* attack, where they emerged as prominent defence-related transcripts in microarray analyses (Martel et al., 2015), reinforcing their role as key molecular markers in plant responses to mite herbivory.

The putative *glutamate receptor-like* (*GLR*) gene was strongly upregulated in all scion/rootstock combinations, with expression increasing 10-fold in ‘MA’, 40-fold in ‘C35’ and ‘CI’, and up to 80-fold in ‘CA’. *GLR* proteins play key roles in sensing leaf damage and regulating defence signalling pathways, as well as in wound and pathogen responses (Mousavi et al., 2013; Yan et al., 2024). In mite-infested sour orange, *GLR* overexpression and glutamate accumulation were linked to systemic resistance (Agut et al., 2016). Exogenous glutamate application also primed plants for stronger, faster responses to pests and pathogens, highlighting the role of GLRs in early defence signalling.

**Figure 6:**
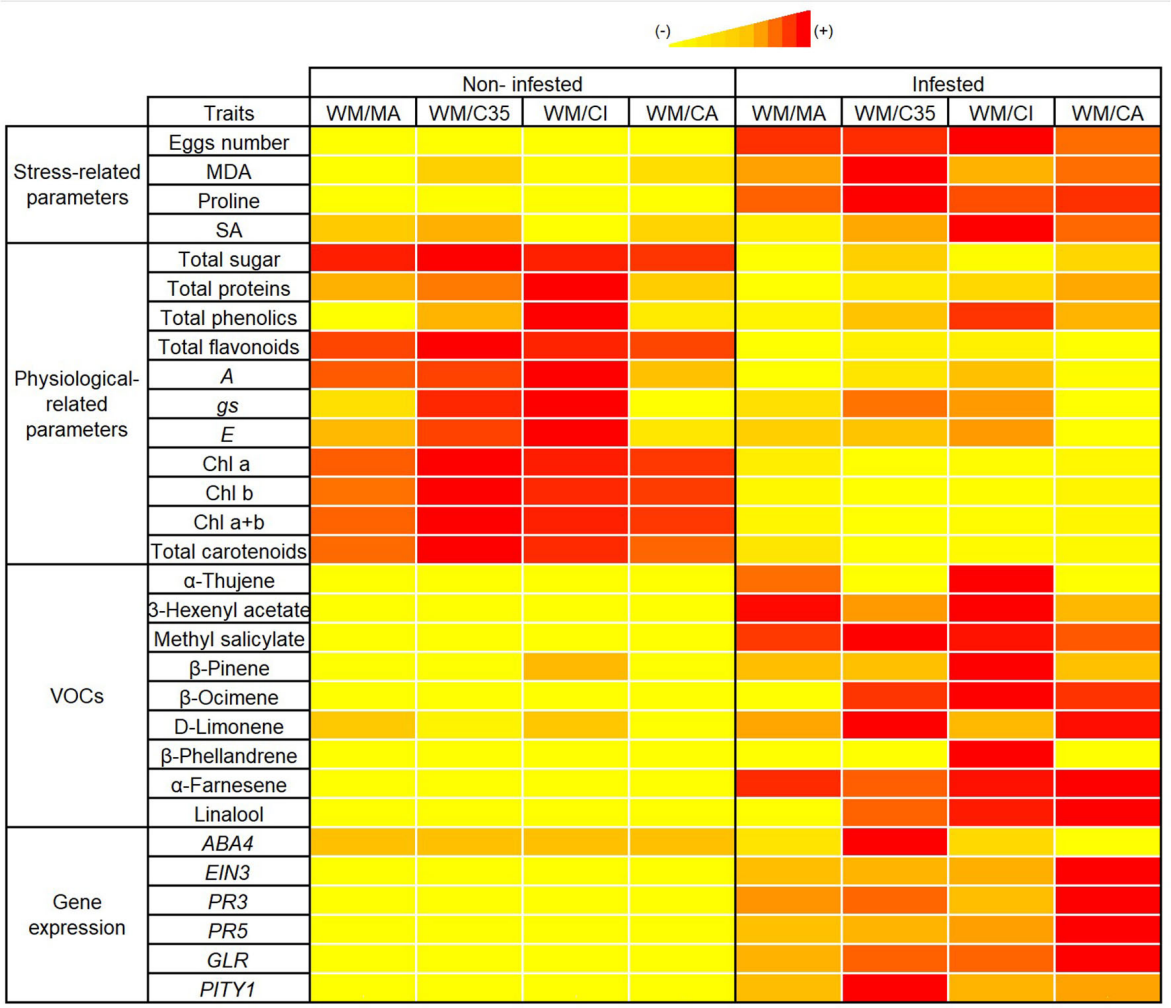
Heatmap showing changes in physiological, biochemical, molecular, and volatile organic compound (VOC) traits in four *Citrus* scion–rootstock combinations, comparing non-infested and *Panonychus citri*-infested plants. Combinations include ‘WM/MA’ ('Macrophylla', *Citrus macrophylla* Wester), ‘WM/C35’ [*C. sinensis* × *P. trifoliata* (South African)], ‘WM/CI’ [‘Citrumelo’ (*Citrus paradisi* Macf. ‘Duncan’ grapefruit × *P. trifoliata*)], and ‘WM/CA’ ['Carrizo citrange' (*Citrus sinensis* (L.) Osbeck × *Poncirus trifoliata* (L.) Raf.)], each grafted with ‘W. Murcott’ (WM) as the scion. Traits are grouped into stress-related parameters, physiological parameters, VOCs, and defense-related gene expression. Color intensity represents the relative value of each trait within the non-infested vs. infested comparison for each combination, with red indicating the highest value, followed by orange and yellow, reflecting decreasing relative levels. Parameters include malondialdehyde (MDA), proline, salicylic acid (SA), photosynthesis (*A*), stomatal conductance (*gs*), transpiration (*E*), chlorophyll (Chl a, Chl b, Chl a+b), carotenoids, selected VOCs (e.g., α-thujene, β-pinene, methyl salicylate), and defense-related gene expression (*ABA4*, *EIN3*, *PR3*, *PR5*, *GLR*, *PITY1*). Each trait was independently normalized using min–max scaling across both infestation conditions for a given combination, allowing comparison of within-combination variation. This multivariate representation provides an integrated overview of rootstock-modulated responses to red mite infestation.

In conclusion, *Panonychus citri* herbivory amplifies rootstock-driven differences in the physiological, biochemical, and molecular responses of ‘W. Murcott’ mandarin scions under semi-field conditions. Rootstocks significantly influenced stress markers—including MDA, proline, SA, soluble sugars, and proteins—as well as VOC emission profiles, indicating modulation of both primary and secondary metabolism. Among the combinations tested, ‘WM/CI’ and ‘WM/CA’ emerged as promising rootstocks for enhancing scion performance and red mite tolerance. ‘WM/CI’ showed the lowest MDA levels and highest accumulation of defence-related metabolites, while ‘WM/CA’ promoted reprogramming of defence genes, including *ABA4* suppression. The consistently higher expression of *PR5* over *PR3* across combinations support a predominantly SA-mediated defence response. These integrated responses are visually summarized in the heatmap (**Figure 6**), which highlights distinct biochemical and transcriptional patterns across scion/rootstock combinations induced by infestation. Both ‘WM/CI’ and ‘WM/CA’ showed increased VOC emission (e.g., β-pinene, MeSA, β-ocimene) and upregulated *PR5* and *GLR* expression, suggesting strong inducible defenses. In contrast, ‘WM/MA’ displayed limited changes in stress markers and gene expression, indicating weaker inducible responses due to red mite attack. Meanwhile, ‘WM/C35’ was distinguished by its high MDA accumulation and strong induction of *PITY1*, pointing to more pronounced oxidative stress and activation of damage-related pathways.

Despite extensive research on ungrafted citrus rootstocks and abiotic stress (Simpson et al., 2014; Long et al., 2017; Huang et al., 2020; Arjona-López et al., 2023), few studies have addressed how rootstocks modulate scion responses to herbivory (Agut et al., 2016; Shaltiel-Harpaz et al., 2018). Our findings underscore the pivotal role of rootstock selection in shaping scion resilience under biotic stress, through coordinated changes in metabolite profiles and gene expression. Future research should explore the functional roles of key metabolites and regulatory genes, and evaluate resistance priming through exogenous hormone applications. Ultimately, integrating multi-level markers - from metabolic to transcriptional -offers a robust framework for rootstock selection in breeding and nursery programmes aimed at developing citrus cultivars resilient to evolving agroecological challenges.

## Data Availability

The original contributions presented in the study are publicly available. This data can be found here: https://github.com/TOMMYRIOJA/data-repository-publication-citrus-Panonychus-citri.
