# Supplementary material for: Red mite (Panonychus citri) attack amplifies citrus rootstock-driven responses in physiological and biochemical traits, VOC emission, and expression of defence-related genes in mandarin scions
Source: Front Plant Sci. 2025 Sep 4;16:1645535. doi: 10.3389/fpls.2025.1645535 (PMC12443807; doi:10.3389/fpls.2025.1645535)
Supplement: Supplementary file 1 [file DataSheet1.docx]

Supplementary figure


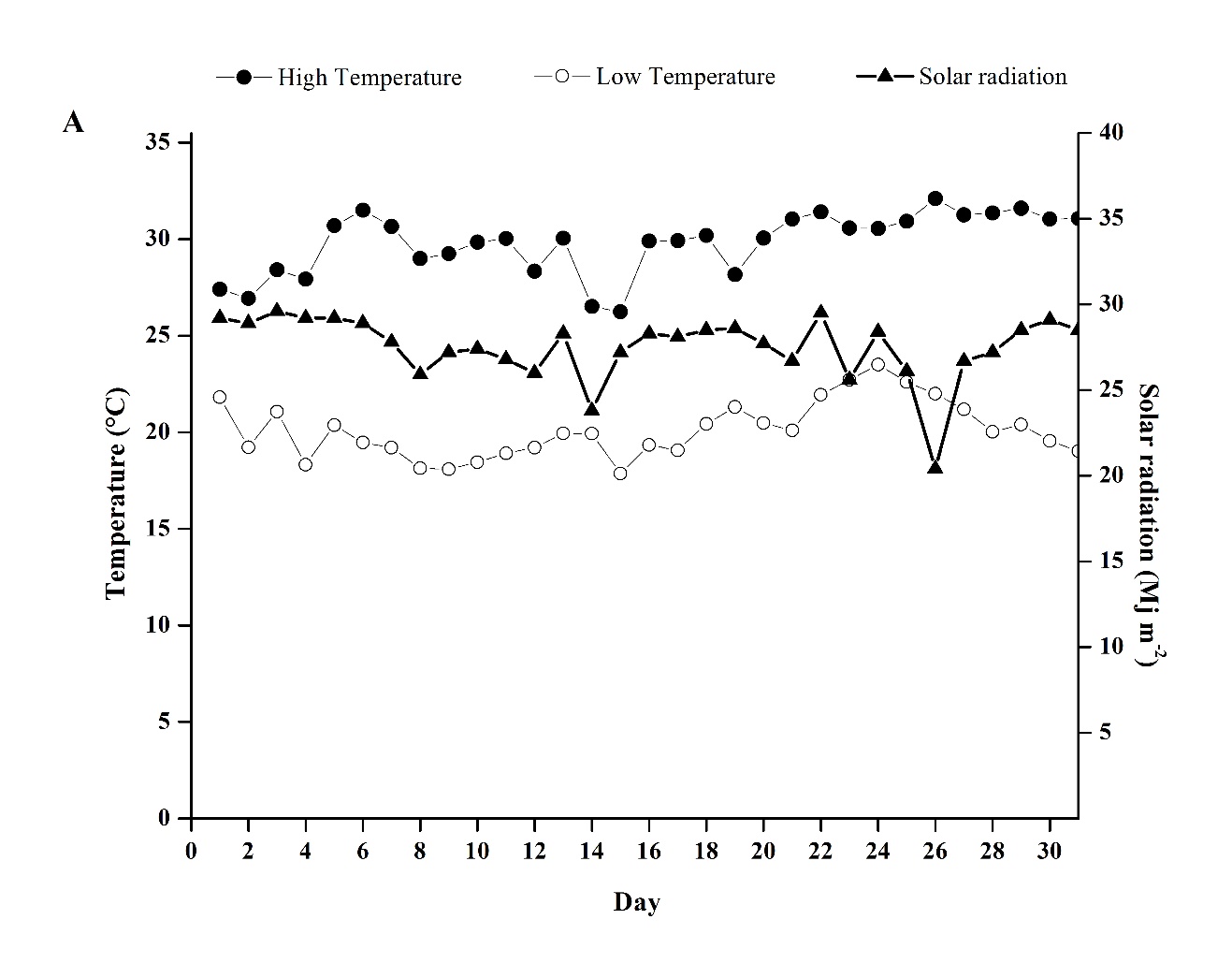


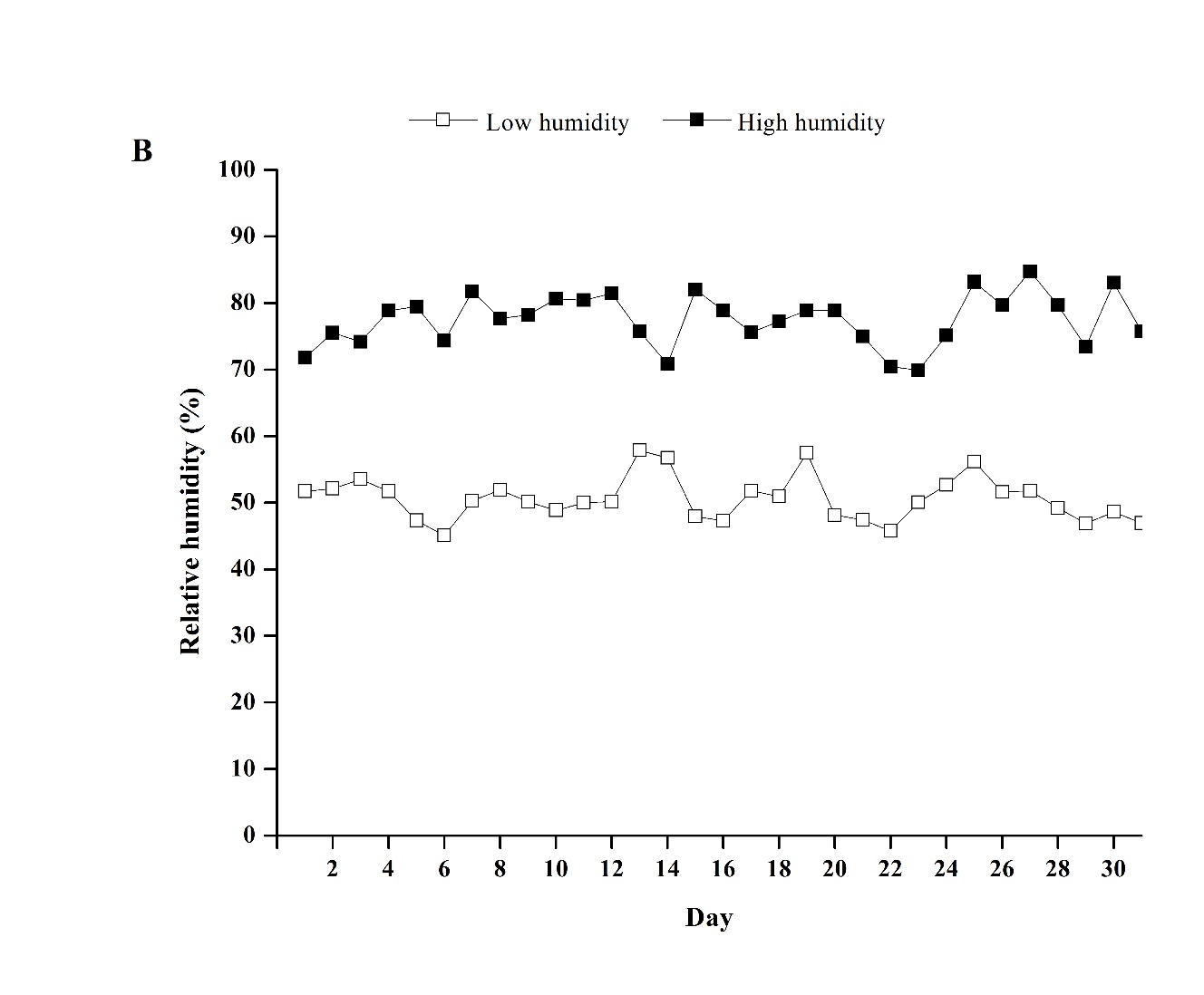


**Figure S1.** Meteorological data registered in January 2022. **(A)** The solar radiation data was collected from the weather station at Universidad Arturo Prat. The temperature **(A)** and humidity **(B)** were monitored inside the greenhouses using the digital thermo-hygrometer RC-4HC.


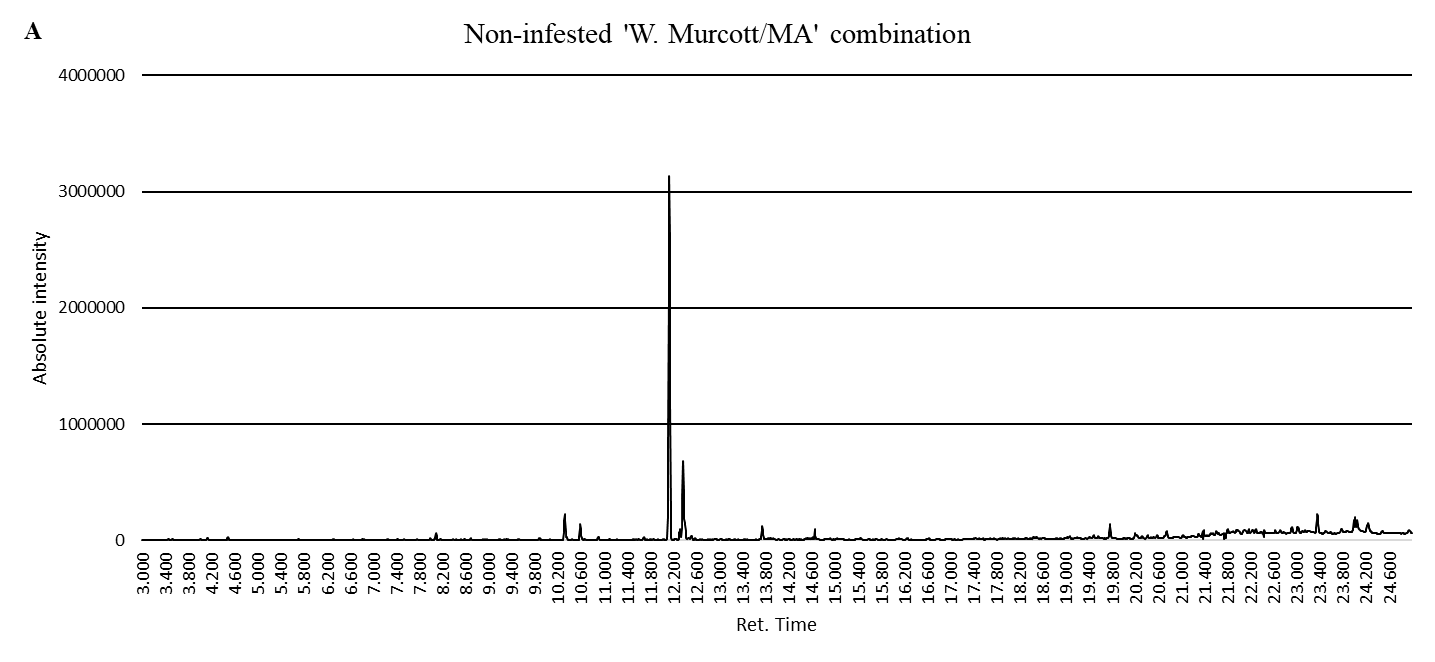


**A:** Description [retention time (compound)]: 3.452 (3-Hexanone); 3.521 (2-Hexanone); 4.129 (2,3,3-trimethylhexane); 4.486 (2,4-dimethyl-1-heptene); 8.088 (D-limonene); 10.900 (tridecane); 12.134 (1-(4-ethylphenyl)-ethanone); 12.371 (Pentadecane); 13.742 (Tetradecane); 19.766 (2,2,4-trimethyldecane); 23.360 (2,2-dimethyleicosane.


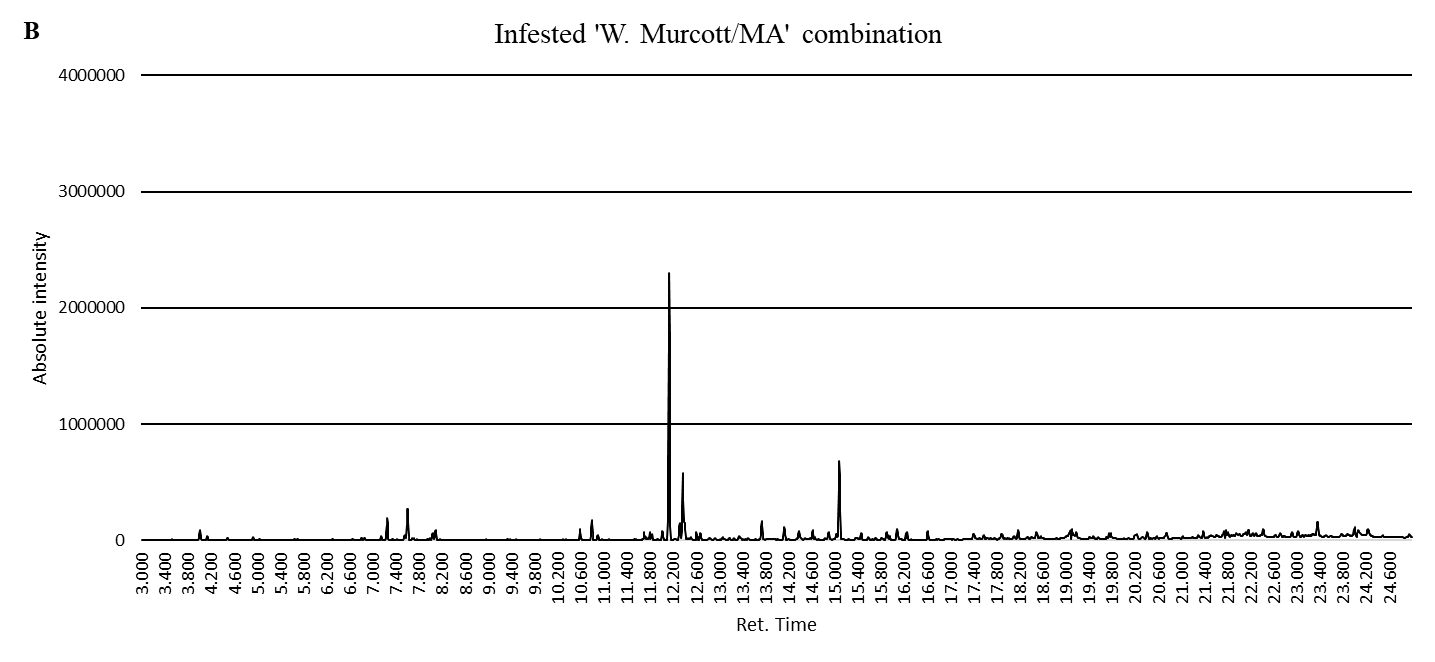


**B:** Description [retention time (compound)]: 3.458 (3-hexanone); 3.528 (2-hexanone); 4.133 (2,3,3-trimethylhexane); 4.490 (2,4-dimethyl-1-heptene); 6.308 (α-thujene); 7.150 (β-pinene); 7.558 (6-ethyl-2-methyldecane); 7.605 (3-hexenyl acetate); 8.039 (D-limonene); 10.794 (methyl salicylate); 10.901 (tridecane); 12.134 (1-(4ethylphenyl)-ethanone); 12.372 (tetradecane); 13.742 (1,2,3,6-tetramethylbicyclo[2.2.2]octa-2,5-diene); 14.126 (α-farnesene); 15.085 (hexadecane); 16.246 (pentadecane); 18.181 (2,2-dimethyleicosane).


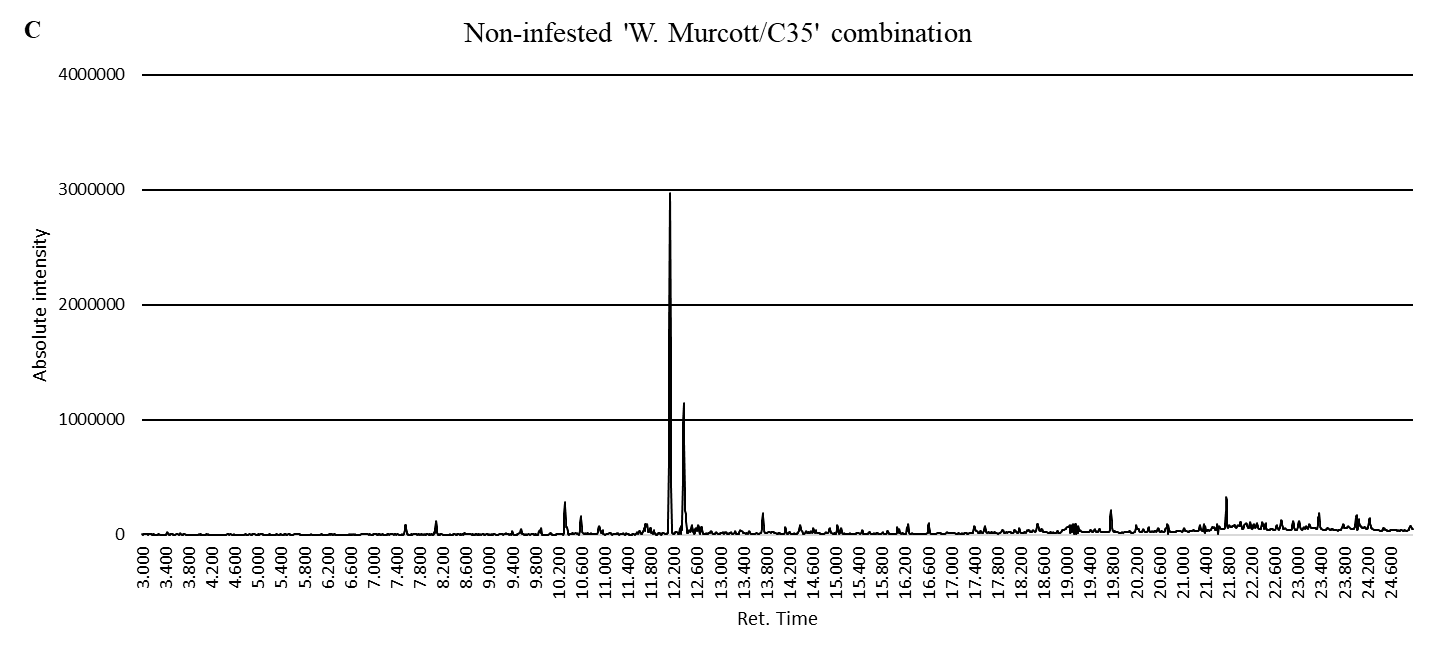


**C:** Description [retention time (compound)]: 3.435 (3-hexanone); 3.660 (3-hexanol); 7.555 (6-ethyl-2-methyldecane); 8.088 (D-limonene); 10.318 (3-ethylbenzaldehyde); 10.593 (4-ethylbenzaldehyde); 10.903 (tridecane); 12.137 (1-(4-ethylphenyl)-ethanone); 12.373 (pentadecane); 13.743 (tetradecane); 19.768 (2,2,4-trimethyldecane); 21.761 (2,2-dimethyleicosane).


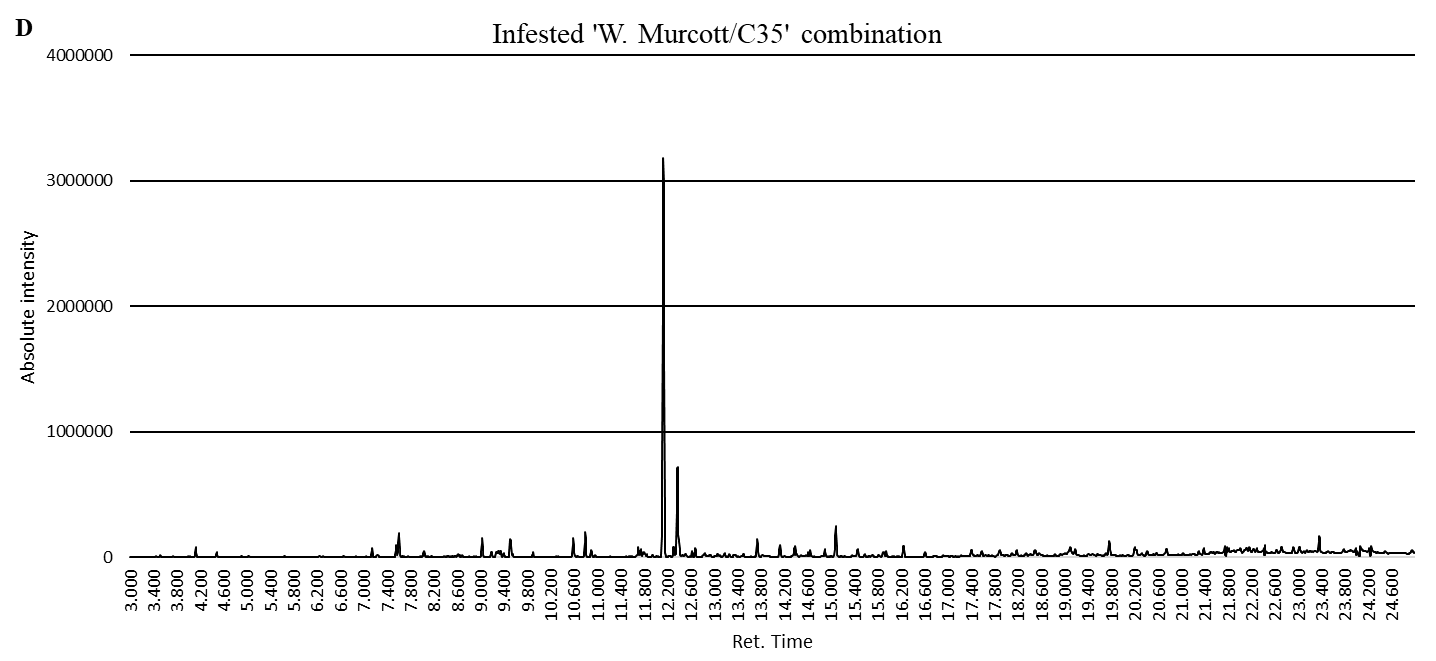


**D:** Description [retention time (compound)]: 3.446 (3-hexanone); 3.516 (2-hexanone); 4.125 (2,3,3-trimethylhexane); 7.051 (β-pinene); 7.146 (6-ethyl-2-methyldecane); 7.554 (3-hexenyl acetate); 7.602 (β-ocimene); 8.036 (D-limonene); 9.031 (linalool); 10.591 (4-ethylbenzaldehyde); 10.793 (methyl salicylate); 10.900 (tridecane); 12.136 (1-(4-ethylphenyl)-ethanone); 12.371 (tetradecane); 13.742 (1,2,3,6-tetramethylbicyclo[2.2.2]octa-2,5-diene); 14.128 (α-farnesene); 15.084 (hexadecane); 16.246 (pentadecane); 23.360 (2,2-dimethyleicosane).


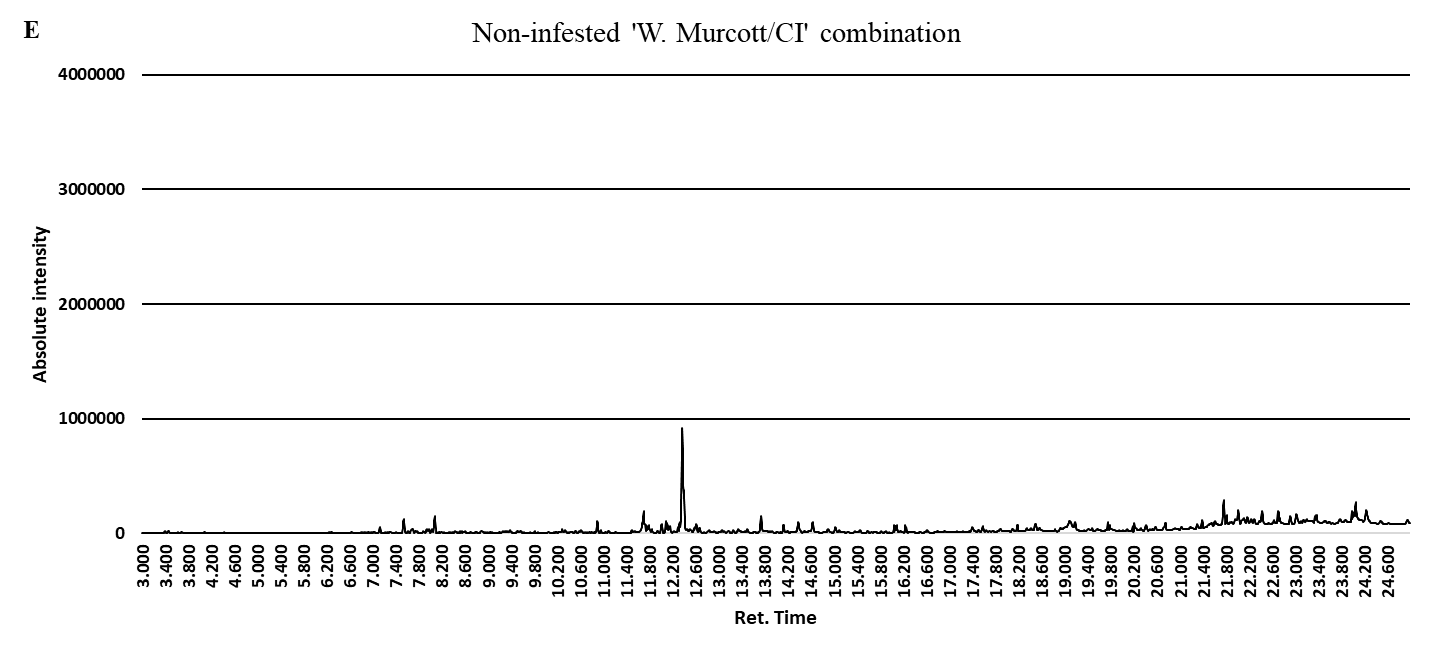


**E:** Description [retention time (compound)]: 3.394 (3-hexanone); 3.464 (2-hexanone); 3.621 (3-hexanol); 7.128 (β-pinene); 7.540 (6-ethyl-2-methyldecane); 8.074 (D-limonene); 10.894 (tridecane); 12.367 (pentadecane); 13.738 (tetradecane); 21.758 (2,2-dimethyleicosane).


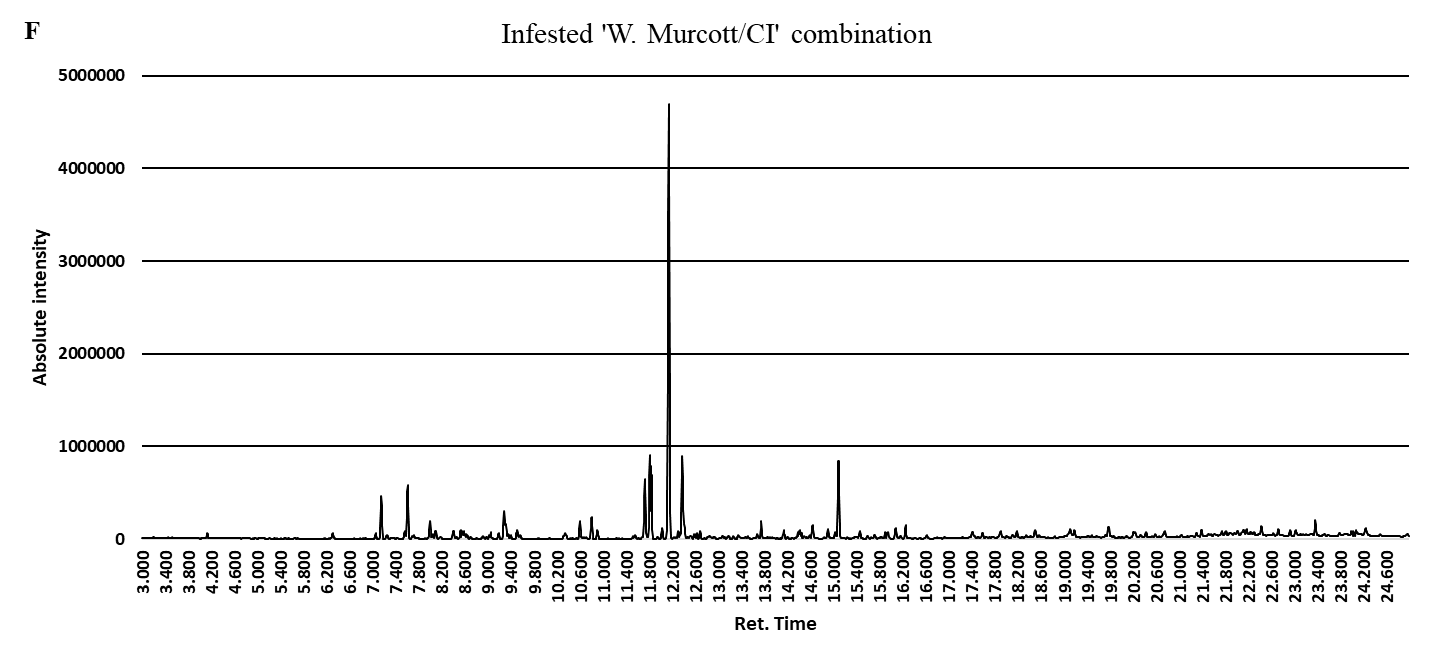


**F:** Description [retention time (compound)]: 3.449 (3-hexanone); 3.519 (2-hexanone); 4.127 (2,3,3-trimethlyhexane); 6.308 (α-thujene); 7.053 (β-phellandrene); 7.150 (β-pinene); 7.558 (6-ethyl-2-methyldecane); 7.606 (3-hexenyl acetate); 7.995 (β-ocimene); 8.042 (D-limonene); 9.279 (linalool); 10.594 (4-ethylbenzaldehyde); 10.797 (methyl salicylate); 10.902 (tridecane);12.023 (1-(4-ethylphenyl)-ethanone); 12.142 (tetradecane); 12.375 (1,2,3,6-tetramethylbicyclo[2.2.2]octa-2,5-diene); 13.745 (α-farnesene); 15.088 (hexadecane); 16.249 (pentadecane); 23.365 (2,2-dimethyleicosane).


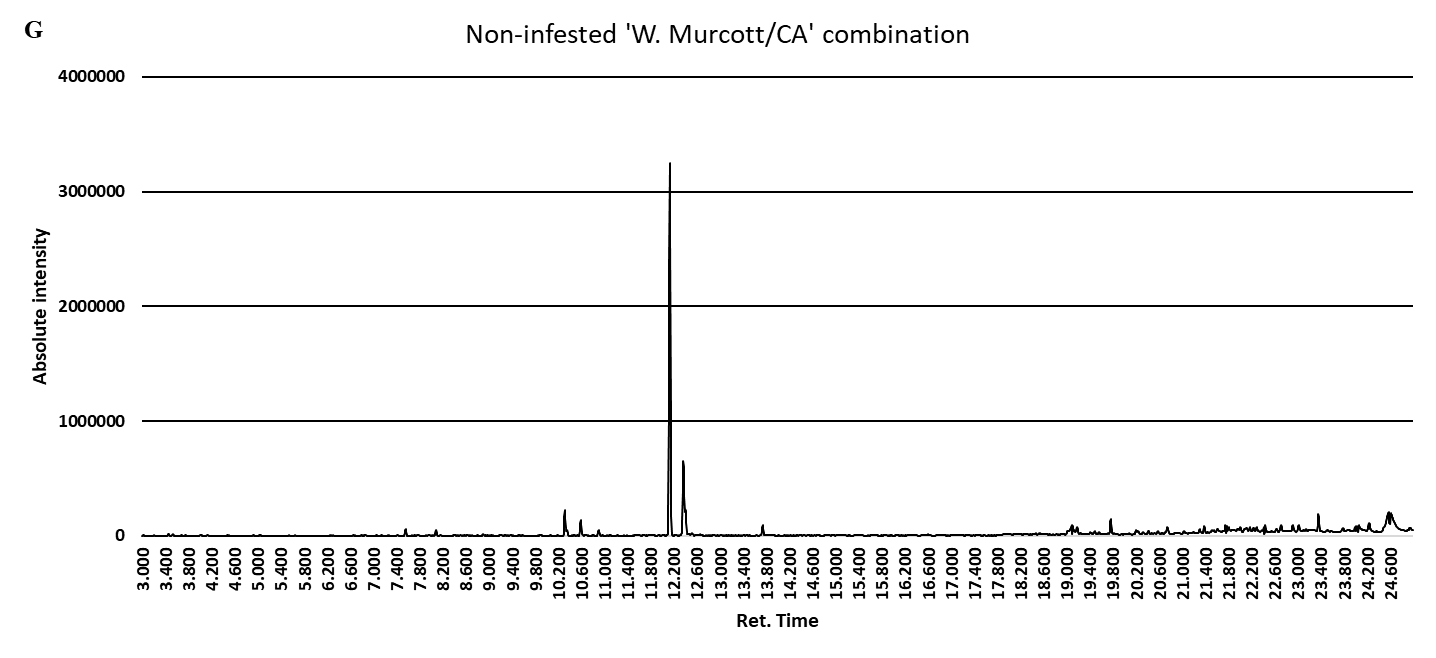


**G:** Description [retention time (compound)]: 3.454 (3-hexanone); 3.525 (2-hexanone); 7.555 (6-ethyl-2-methyldecane); 8.087 (D-limonene); 10.316 (3-ethylbenzaldehyde); 10.591 (4-ethylbenzaldehyde); 10.898 (tridecane); 12.134 (1-(4-ethylphenyl)-ethanone); 12.369 (pentadecane); 13.740 (tetradecane); 19.765 (2,2,4-trimethyldecane); 23.359 (2,2-dimethyleicosane).


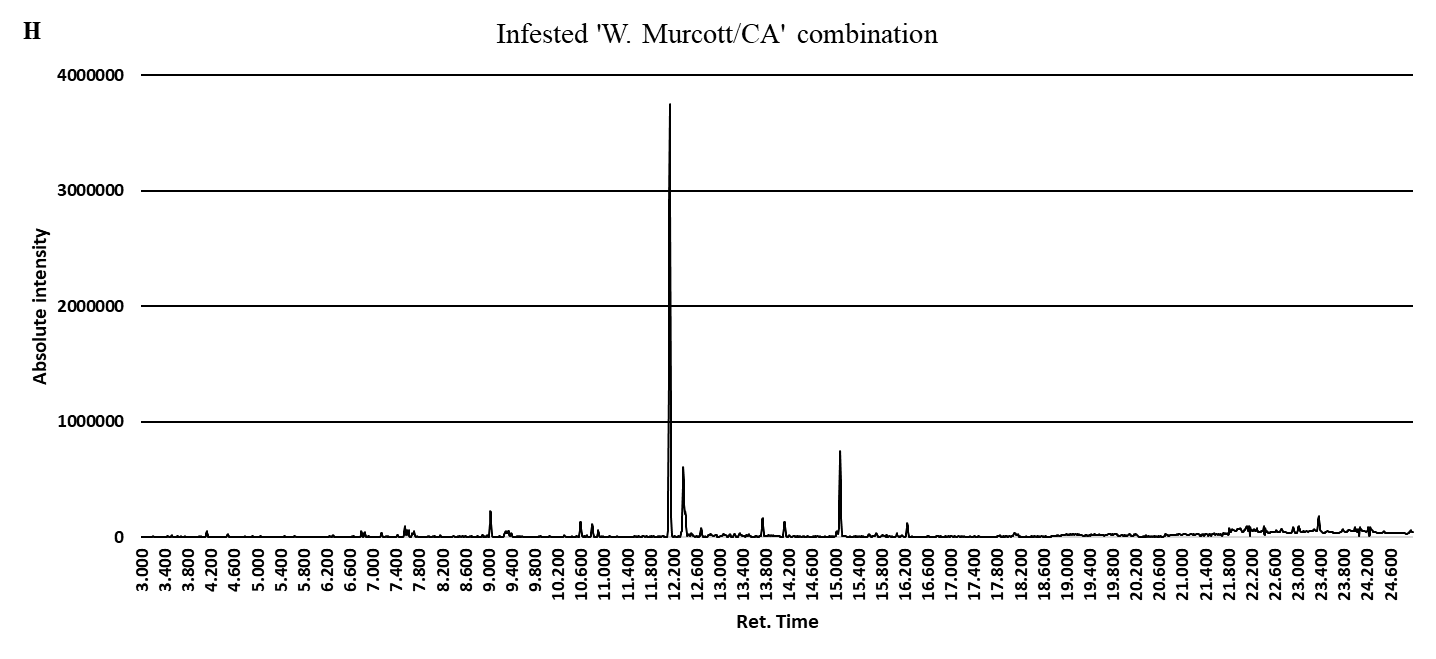


**H:** Description [retention time (compound)]: 3.458 (3-hexanone); 3.526 (2-hexanone); 4.134 (2,3,3-trimethylhexane); 4.491 (2,4-dimethyl-1-heptene); 6.312 (β-pinene); 7.058 (6-ethyl-2-methyldecane); 7.154 (3-hexenyl acetate); 7.560 (β-ocimene); 7.609 (D-limonene); 9.037 (linalool); 10.596 (4-ethylbenzaldehyde); 10.799 (methyl salicylate); 10.903 (tridecane)12.141 (1-(4-ethylphenyl)-ethanone); 12.376 (tetradecane); 13.747 (1,2,3,6-tetramethylbicyclo[2.2.2]octa-2,5-diene); 14.130 (α-farnesene); 15.089 (hexadecane); 16.250 (pentadecane); 23.366 (2,2-dimethyleicosane).

**Figure S2.** VOC identification by using LabSolutions GCMS software (v4.30, Shimadzu) and the NIST library (version 2.0). Sample (1 µ-L) of each infestation level, and scion/rootstock combination, which were injected in splitless mode into a gas chromatograph coupled to a mass spectrometer (GC-MS; QP2010 Ultra, Shimadzu, Kyoto, Japan) equipped with an RTx5 capillary column (30 m, 0.25 mm internal diameter, 0.25 µm film thickness; Restek, Bellefonte, Pennsylvania, USA). Chromatograms from non-infested and infested mandarin of each scion/rootstock combination are showed: (**A-B**) ‘W. Murcott/Macrophylla’; (**C-D**) ‘W. Murcott/C35’; (**E-F**) ‘W. Murcott/Citrumelo’; and (**G-H**) ‘W. Murcott/Carrizo’.

**
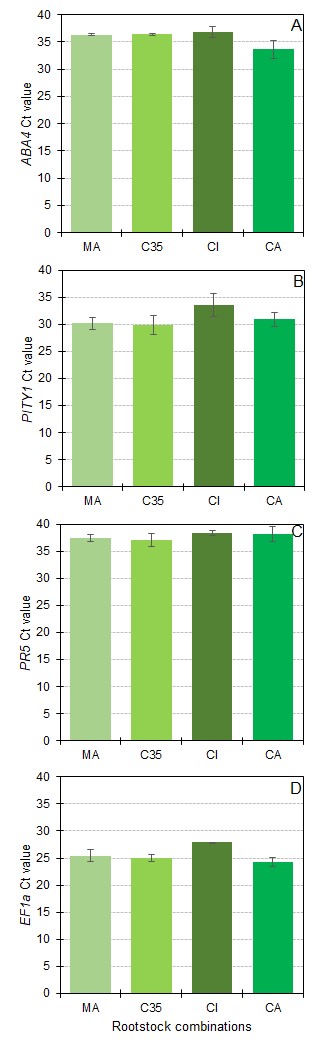
Figure S3.** Ct values of target genes (A: *ABA4*, B: *PITY*, C: *PR5*) and the housekeeping gene *EF1α* (D) in non-infested control plants grafted onto four different citrus rootstocks: MA, C35, CI, and CA. Bars represent mean Ct values ± SE. These values were used to calculate ΔCt and subsequently ΔΔCt for gene expression analyses. Each bar reflects the average of biological replicates per rootstock, confirming that *CNT* corresponds to rootstock-specific non-infested controls. Transcripts for *PR3*, *GLR*, and *EIN3* showed highly variable and consistently high Ct values (ranging from 38 to 40) across rootstocks, indicating unreliable detection; therefore, they were excluded from downstream analyses.


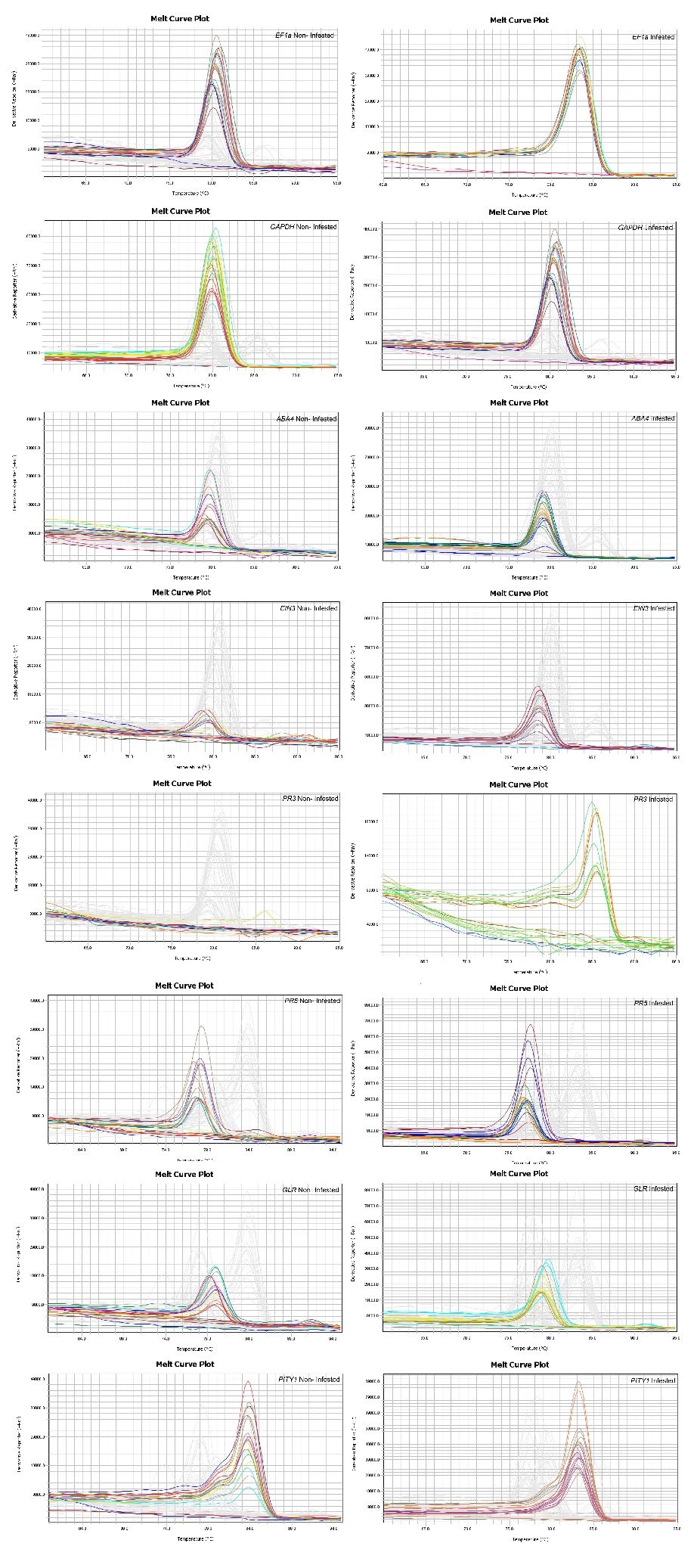


**Figure S4.** Melt curve plots of qRT-PCR amplicons for reference and target genes in citrus scion/rootstock combinations under control (non-infested) and *Panonychus citri* - infested (Infested) conditions. Each panel displays the dissociation (melt) curve for a specific gene: *EF1α*, *GAPDH* (reference genes); *ABA4*, *EIN3*, *PR3*, *PR5*, *GLR*, and *PITY1* (target genes). Melt curves were generated using a QuantStudio 3 Real-Time PCR System and analyzed with QuantStudio Design & Analysis Desktop Software v1.5.1 (Applied Biosystems by Thermo Fisher Scientific) to assess the specificity of each qRT-PCR amplicon. A single, sharp peak per sample indicates specific amplification without primer-dimer or non-specific artifacts. Non-Infested and Infested samples are shown on the left and right columns, respectively. *PR3* expression was very low and inconsistent across the replicates, particularly in control samples, for this reason, CT values of *PR3* in control samples were excluded from downstream expression analysis due to their poor amplification reliability. Overall, melt profiles for most genes confirm amplification specificity and consistency across treatments.
