# Supplementary material for: Red mite (Panonychus citri) attack amplifies citrus rootstock-driven responses in physiological and biochemical traits, VOC emission, and expression of defence-related genes in mandarin scions
Source: Front Plant Sci. 2025 Sep 4;16:1645535. doi: 10.3389/fpls.2025.1645535 (PMC12443807; doi:10.3389/fpls.2025.1645535)
Supplement: Supplementary file 2 [file DataSheet2.docx]

Supplementary table

**Table S1.** Primers used for qPCR analysis of gene expression. Primer sequences were published in Agut et al. (2014), and Agut et al., (2016). The primer efficiency fluctuates between 94 and 100%.

| ID | Accession | Forward primer 5' → 3' | Reverse primer 5' → 3' | Amplicon length (bp) | Tm |
| --- | --- | --- | --- | --- | --- |
| *PR-3* | Cit.1727.1.S1_s_at | CTGGACATTTGTGCTACATAGAAG | CACAAGGCAGTCTTAAATGACA | 229 | 60 |
| *EIN3* | Cit.5616.1.S1_s_at | AGGTGTGAGGCTTCTTCCTT | AAAGCAGTTTTGGACATTGG | 187 | 60 |
| *ABA4* | Cit.318.1.S1_s_at | CACTCATGGTTTTTGCTCCT | AGCTCTGGGAGCCAGTATTT | 163 | 60 |
| *PR-5* | BAI63297.1 | CATCAAGCTTCACAGTGCTTAG | CCACAACGTACAGACTGATGAC | 152 | 60 |
| *PI TYPE1* | DR908940 | CTTTCTCTTTGCCTTTGCTG | CCGTTTACACCAACCAGTTC | 168 | 60 |
| *GLR* | XM_024179289 | GGGGCGGGACATTAAATCTT | CTGCGGATACCCATGTTCAA | 179 | 60 |

**Table S2.** List of compounds. Sample (1µ-L) injected in splitless mode into a gas chromatograph coupled to a mass spectrometer (GC-MS; QP2010 Ultra, Shimadzu, Kyoto, Japan) equipped with an RTx5 capillary column (30 m, 0.25 mm internal diameter, 0.25 µm film thickness; Restek, Bellefonte, Pennsylvania, USA). VOCs collected from non-infested ‘W. Murcott’ mandarin. Plants grafted onto ‘Macrophylla’ rootstock (‘WM/MA’). Quantification was conducted using the internal standard method, with tridecane (Sigma-Aldrich) as the analytical standard.

| **Ret, Time** | **Area** | **Height** | **A/H** | **ug** | **ug mL^-1^** | **Compound** |
| --- | --- | --- | --- | --- | --- | --- |
| 3.452 | 19824 | 13698 | 1,45 | 1,0 | 4,9 | 3-Hexanone |
| 3.521 | 23536 | 14569 | 1,62 | 1,2 | 5,8 | 2-Hexanone |
| 4.129 | 34502 | 22928 | 1,51 | 1,7 | 8,5 | 2,3,3-Trimethylhexane |
| 4.486 | 42615 | 29881 | 1,43 | 2,1 | 10,5 | 2,4-dimethyl-1-Heptene |
| 8.088 | 86273 | 59350 | 1,46 | 4,2 | 21,2 | D-Limonene |
| 10.316 | 317513 | 211546 | 1,49 | 15,6 | 78,1 | 3-Ethylbenzaldehyde |
| 10.591 | 196698 | 132278 | 1,48 | 9,7 | 48,4 | 4-Ethylbenzaldehyde |
| 10.900 | 61006 | 31874 | 1,91 | 3,0 | 15,0 | Tridecane* |
| 12.134 | 5152932 | 3120190 | 1,65 | 253,4 | 1267,0 | 1-(4-ethylphenyl)-Ethanone |
| 12.371 | 986990 | 600078 | 1,62 | 48,5 | 242,7 | Pentadecane |
| 13.742 | 152624 | 105922 | 1,44 | 7,5 | 37,5 | Tetradecane |
| 19.766 | 207909 | 116037 | 1,79 | 10,2 | 51,1 | 2,2,4-Trimethyldecane |
| 23.360 | 296127 | 162776 | 1,82 | 14,6 | 72,8 | 2,2-Dimethyleicosane |

*Internal standard.

**Table S3.** List of compounds. Sample (1µ-L) injected in splitless mode into a gas chromatograph coupled to a mass spectrometer (GC-MS; QP2010 Ultra, Shimadzu, Kyoto, Japan) equipped with an RTx5 capillary column (30 m, 0.25 mm internal diameter, 0.25 µm film thickness; Restek, Bellefonte, Pennsylvania, USA). VOCs collected from infested ‘W. Murcott’ mandarin. Plants grafted onto ‘Macrophylla’ rootstock (‘WM/MA’). Quantification was conducted using the internal standard method, with tridecane (Sigma-Aldrich) as the analytical standard.

| **Ret, Time** | **Area** | **Height** | **A/H** | **ug** | **ug mL^-1^** | **Compound** |
| --- | --- | --- | --- | --- | --- | --- |
| 3.458 | 8832 | 6416 | 1,38 | 0,4 | 1,8 | 3-Hexanone |
| 3.528 | 6287 | 7021 | 0,9 | 0,3 | 1,3 | 2-Hexanone |
| 4.133 | 55950 | 39299 | 1,42 | 2,3 | 11,3 | 2,3,3-Trimethylhexane |
| 4.490 | 26051 | 16874 | 1,54 | 1,0 | 5,2 | 2,4-dimethyl-1-Heptene |
| 6.308 | 9780 | 9094 | 1,08 | 0,4 | 2,0 | alpha-Thujene |
| 7.150 | 34273 | 30830 | 1,11 | 1,4 | 6,9 | beta-Pinene |
| 7.558 | 63989 | 39953 | 1,6 | 2,6 | 12,9 | 6-Ethyl-2-methyldecane |
| 7.605 | 451354 | 269759 | 1,67 | 18,2 | 90,8 | 3-Hexenyl acetate |
| 8.039 | 81396 | 54581 | 1,49 | 3,3 | 16,4 | D-Limonene |
| 10.593 | 136310 | 91005 | 1,5 | 5,5 | 27,4 | 4-Ethylbenzaldehyde |
| 10.794 | 261448 | 166740 | 1,57 | 10,5 | 52,6 | Methyl salicylate |
| 10.901 | 74557 | 44293 | 1,68 | 3,0 | 15,0 | Tridecane* |
| 12.134 | 3699178 | 2291111 | 1,61 | 148,8 | 744,2 | 1-(4-ethylphenyl)-Ethanone |
| 12.372 | 1199449 | 568643 | 2,11 | 48,3 | 241,3 | Tetradecane |
| 13.742 | 232719 | 153324 | 1,52 | 9,4 | 46,8 | 1,2,3,6-Tetramethylbicyclo[2.2.2]octa-2,5-diene |
| 14.126 | 203524 | 112921 | 1,8 | 8,2 | 40,9 | alpha-Farnesene |
| 15.085 | 1023448 | 659928 | 1,55 | 41,2 | 205,9 | Hexadecane |
| 16.246 | 189373 | 115055 | 1,65 | 7,6 | 38,1 | Pentadecane |
| 18.181 | 117392 | 74429 | 1,58 | 4,7 | 23,6 | 3-Methylheptadecane |
| 23.362 | 205909 | 114800 | 1,79 | 8,3 | 41,4 | 2,2-Dimethyleicosane |

*Internal standard.

**Table S4.** List of compounds. Sample (1µ-L) injected in splitless mode into a gas chromatograph coupled to a mass spectrometer (GC-MS; QP2010 Ultra, Shimadzu, Kyoto, Japan) equipped with an RTx5 capillary column (30 m, 0.25 mm internal diameter, 0.25 µm film thickness; Restek, Bellefonte, Pennsylvania, USA). VOCs collected from non-infested ‘W. Murcott’ mandarin. Plants grafted onto ‘C35’ rootstock (‘WM/C35’). Quantification was conducted using the internal standard method, with tridecane (Sigma-Aldrich) as the analytical standard.

| **Ret, Time** | **Area** | **Height** | **A/H** | **ug** | **ug mL^-1^** | **Compound** |
| --- | --- | --- | --- | --- | --- | --- |
| 3.435 | 32378 | 19497 | 1,66 | 0,5 | 2,7 | 3-Hexanone |
| 3.660 | 14941 | 10596 | 1,41 | 0,3 | 1,3 | 3-Hexanol |
| 7.555 | 150273 | 86156 | 1,74 | 2,5 | 12,7 | 6-Ethyl-2-methyldecane |
| 8.088 | 178055 | 114329 | 1,56 | 3,0 | 15,0 | D-Limonene |
| 10.318 | 520286 | 283769 | 1,83 | 8,8 | 43,9 | 3-Ethylbenzaldehyde |
| 10.593 | 259919 | 162481 | 1,6 | 4,4 | 21,9 | 4-Ethylbenzaldehyde |
| 10.903 | 177679 | 74944 | 2,37 | 3,0 | 15,0 | Tridecane* |
| 12.137 | 5194244 | 2956859 | 1,76 | 87,7 | 438,5 | 1-(4-ethylphenyl)-Ethanone |
| 12.373 | 1966596 | 1106925 | 1,78 | 33,2 | 166,0 | Pentadecane |
| 13.743 | 260517 | 168560 | 1,55 | 4,4 | 22,0 | Tetradecane |
| 19.768 | 347176 | 190461 | 1,82 | 5,9 | 29,3 | 2,2,4-Trimethyldecane |
| 23.364 | 379598 | 204321 | 1,86 | 6,4 | 32,0 | 2,2-Dimethyleicosane |

*Internal standard.

**Table S5.** List of compounds. Sample (1µ-L) injected in splitless mode into a gas chromatograph coupled to a mass spectrometer (GC-MS; QP2010 Ultra, Shimadzu, Kyoto, Japan) equipped with an RTx5 capillary column (30 m, 0.25 mm internal diameter, 0.25 µm film thickness; Restek, Bellefonte, Pennsylvania, USA). VOCs collected from infested ‘W. Murcott’ mandarin. Plants grafted onto ‘C35’ rootstock (‘WM/C35’). Quantification was conducted using the internal standard method, with tridecane (Sigma-Aldrich) as the analytical standard.

| **Ret, Time** | **Area** | **Height** | **A/H** | **ug** | **ug mL^-1^** | **Compound** |
| --- | --- | --- | --- | --- | --- | --- |
| 3.446 | 15310 | 11085,0 | 1,38 | 0,5 | 2,4 | 3-Hexanone |
| 3.516 | 24562,0 | 14779,0 | 1,66 | 0,8 | 3,8 | 2-Hexanone |
| 4.125 | 76678 | 52488,0 | 1,46 | 2,4 | 11,9 | 2,3,3-Trimethylhexane |
| 7.051 | 4412 | 4973,0 | 0,89 | 0,1 | 0,7 | β-Pinene |
| 7.146 | 90751 | 57318,0 | 1,58 | 2,8 | 14,1 | 6-Ethyl-2-methyldecane |
| 7.554 | 98527 | 56774,0 | 1,74 | 3,1 | 15,3 | 3-Hexenyl acetate |
| 7.602 | 300573 | 193480,0 | 1,55 | 9,3 | 46,6 | β-ocimene |
| 8.036 | 65853 | 46536,0 | 1,42 | 2,0 | 10,2 | D-Limonene |
| 9.031 | 198114 | 131282,0 | 1,51 | 6,1 | 30,7 | Linalool |
| 10.591 | 221146 | 135581,0 | 1,63 | 6,9 | 34,3 | 4-Ethylbenzaldehyde |
| 10.793 | 301909 | 188709,0 | 1,6 | 9,4 | 46,8 | Methyl salicylate |
| 10.900 | 96809 | 57104,0 | 1,69 | 3,0 | 15,0 | Tridecane* |
| 12.136 | 5473638 | 3172781,0 | 1,73 | 169,6 | 848,1 | 1-(4-ethylphenyl)-Ethanone |
| 12.371 | 1326453 | 697161,0 | 1,9 | 41,1 | 205,5 | Tetradecane |
| 13.742 | 222457 | 140879 | 1,58 | 6,9 | 34,5 | 1,2,3,6-Tetramethylbicyclo[2.2.2]octa-2,5-diene |
| 14.128 | 174067 | 99066 | 1,76 | 5,4 | 27,0 | α-Farnesene |
| 15.084 | 349096 | 234947 | 1,49 | 10,8 | 54,1 | Hexadecane |
| 16.246 | 147032 | 87227 | 1,69 | 4,6 | 22,8 | Pentadecane |
| 23.360 | 228572 | 123966 | 1,84 | 7,1 | 35,4 | 2,2-Dimethyleicosane |

*Internal standard.

**Table S6.** List of compounds. Sample (1µ-L) injected in splitless mode into a gas chromatograph coupled to a mass spectrometer (GC-MS; QP2010 Ultra, Shimadzu, Kyoto, Japan) equipped with an RTx5 capillary column (30 m, 0.25 mm internal diameter, 0.25 µm film thickness; Restek, Bellefonte, Pennsylvania, USA). VOCs collected from non-infested ‘W. Murcott’ mandarin. Plants grafted onto ‘Citrumelo’ rootstock (‘WM/CI’). Quantification was conducted using the internal standard method, with tridecane (Sigma-Aldrich) as the analytical standard.

| **Ret, Time** | **Area** | **Height** | **A/H** | **ug** | **ug mL^-1^** | **Compound** |
| --- | --- | --- | --- | --- | --- | --- |
| 3.394 | 29001 | 17647 | 1,64 | 0,6 | 2,8 | 3-Hexanone |
| 3.464 | 40152 | 21838 | 1,84 | 0,8 | 3,9 | 2-Hexanone |
| 3.621 | 16471 | 11516 | 1,43 | 0,3 | 1,6 | 3-Hexanol |
| 7.128 | 79506 | 51747 | 1,54 | 1,5 | 7,6 | β-Pinene |
| 7.540 | 210441 | 126014 | 1,67 | 4,0 | 20,2 | 6-Ethyl-2-methyldecane |
| 8.074 | 204561 | 140419 | 1,46 | 3,9 | 19,6 | D-Limonene |
| 10.894 | 156251 | 96092 | 1,63 | 3,0 | 15,0 | Tridecane* |
| 12.367 | 1049871 | 699925 | 1,46 | 20,2 | 100,8 | Pentadecane |
| 13.738 | 217347 | 135259 | 1,61 | 4,2 | 20,9 | Tetradecane |
| 21.758 | 289197 | 201091 | 1,45 | 5,6 | 27,8 | 2,2-Dimethyleicosane |

*Internal standard.

**Table S7.** List of compounds. Sample (1µ-L) injected in splitless mode into a gas chromatograph coupled to a mass spectrometer (GC-MS; QP2010 Ultra, Shimadzu, Kyoto, Japan) equipped with an RTx5 capillary column (30 m, 0.25 mm internal diameter, 0.25 µm film thickness; Restek, Bellefonte, Pennsylvania, USA). VOCs collected from infested ‘W. Murcott’ mandarin. Plants grafted onto ‘Citrumelo’ rootstock (‘WM/CI’). Quantification was conducted using the internal standard method, with tridecane (Sigma-Aldrich) as the analytical standard.

| **Ret, Time** | **Area** | **Height** | **A/H** | **ug** | **ug mL^-1^** | **Compound** |
| --- | --- | --- | --- | --- | --- | --- |
| 3.449 | 13074 | 8748,0 | 1,49 | 0,3 | 1,3 | 3-Hexanone |
| 3.519 | 16113 | 10969,0 | 1,47 | 0,3 | 1,7 | 2-Hexanone |
| 4.127 | 87337 | 58206,0 | 1,5 | 1,8 | 9,0 | 2,3,3-Trimethylhexane |
| 6.308 | 82753 | 55860,0 | 1,48 | 1,7 | 8,5 | α-Thujene |
| 7.053 | 106353 | 67084,0 | 1,59 | 2,2 | 10,9 | β-Phellandrene |
| 7.150 | 742493 | 464705,0 | 1,6 | 15,3 | 76,3 | β-Pinene |
| 7.558 | 137547 | 89248,0 | 1,54 | 2,8 | 14,1 | 6-Ethyl-2-methyldecane |
| 7.606 | 925527 | 576944,0 | 1,6 | 19,0 | 95,1 | 3-Hexenyl acetate |
| 7.995 | 281061 | 184152,0 | 1,53 | 5,8 | 28,9 | β-ocimene |
| 8.042 | 41841 | 37679,0 | 1,11 | 0,9 | 4,3 | D-Limonene |
| 9.279 | 553308 | 295465,0 | 1,87 | 11,4 | 56,8 | Linalool |
| 10.594 | 322912 | 190929,0 | 1,69 | 6,6 | 33,2 | 4-Ethylbenzaldehyde |
| 10.797 | 399510 | 235370,0 | 1,7 | 8,2 | 41,0 | Methyl salicylate |
| 10.902 | 146016 | 89873,0 | 1,62 | 3,0 | 15,0 | Tridecane* |
| 12.023 | 176904 | 111100,0 | 1,59 | 3,6 | 18,2 | 1-(4-ethylphenyl)-Ethanone |
| 12.142 | 8019424 | 4682079,0 | 1,71 | 164,8 | 823,8 | Tetradecane |
| 12.375 | 1379914 | 849239,0 | 1,62 | 28,4 | 141,8 | 1,2,3,6-Tetramethylbicyclo[2.2.2]octa-2,5-diene |
| 13.745 | 284731 | 180304 | 1,58 | 5,8 | 29,2 | α-Farnesene |
| 15.088 | 1414860 | 828500 | 1,71 | 29,1 | 145,3 | Hexadecane |
| 16.249 | 235624 | 149055 | 1,58 | 4,8 | 24,2 | Pentadecane |
| 23.365 | 266480 | 153610 | 1,73 | 5,5 | 27,4 | 2,2-Dimethyleicosane |

*Internal standard.

**Table S8.** List of compounds. Sample (1µ-L) injected in splitless mode into a gas chromatograph coupled to a mass spectrometer (GC-MS; QP2010 Ultra, Shimadzu, Kyoto, Japan) equipped with an RTx5 capillary column (30 m, 0.25 mm internal diameter, 0.25 µm film thickness; Restek, Bellefonte, Pennsylvania, USA). VOCs collected from non-infested ‘W. Murcott’ mandarin. Plants grafted onto ‘Carrizo’ rootstock (‘WM/CA’). Quantification was conducted using the internal standard method, with tridecane (Sigma-Aldrich) as the analytical standard.

| **Ret, Time** | **Area** | **Height** | **A/H** | **ug** | **ug mL^-1^** | **Compound** |
| --- | --- | --- | --- | --- | --- | --- |
| 3.454 | 14835 | 11008,0 | 1,35 | 0,5 | 2,7 | 3-Hexanone |
| 3.525 | 17798 | 12318,0 | 1,44 | 0,6 | 3,2 | 2-Hexanone |
| 7.555 | 92688 | 57797 | 1,6 | 3,3 | 16,6 | 6-Ethyl-2-methyldecane |
| 8.087 | 77956 | 52952 | 1,47 | 2,8 | 13,9 | D-Limonene |
| 10.316 | 320119 | 206838 | 1,55 | 11,4 | 57,2 | 3-Ethylbenzaldehyde |
| 10.591 | 221126 | 136023 | 1,63 | 7,9 | 39,5 | 4-Ethylbenzaldehyde |
| 10.898 | 83925 | 48602 | 1,73 | 3,0 | 15,0 | Tridecane* |
| 12.134 | 5326935 | 3244970 | 1,64 | 190,4 | 952,1 | 1-(4-ethylphenyl)-Ethanone |
| 12.369 | 1090862 | 625466 | 1,74 | 39,0 | 195,0 | Pentadecane |
| 13.740 | 139662 | 89205 | 1,57 | 5,0 | 25,0 | Tetradecane |
| 19.765 | 226832 | 124333 | 1,82 | 8,1 | 40,5 | 2,2,4-Trimethyldecane |
| 23.359 | 241087 | 142923 | 1,69 | 8,6 | 43,1 | 2,2-Dimethyleicosane |

*Internal standard.

**Table S9.** List of compounds. Sample (1µ-L) injected in splitless mode into a gas chromatograph coupled to a mass spectrometer (GC-MS; QP2010 Ultra, Shimadzu, Kyoto, Japan) equipped with an RTx5 capillary column (30 m, 0.25 mm internal diameter, 0.25 µm film thickness; Restek, Bellefonte, Pennsylvania, USA). VOCs collected from infested ‘W. Murcott’ mandarin. Plants grafted onto ‘Carrizo’ rootstock (‘WM/CA’). Quantification was conducted using the internal standard method, with tridecane (Sigma-Aldrich) as the analytical standard.

| **Ret, Time** | **Area** | **Height** | **A/H** | **ug** | **ug mL^-1^** | **Compound** |
| --- | --- | --- | --- | --- | --- | --- |
| 3.458 | 12738,0 | 10529,0 | 1,21 | 0,4 | 1,8 | 3-Hexanone |
| 3.526 | 18726,0 | 14278,0 | 1,31 | 0,5 | 2,7 | 2-Hexanone |
| 4.134 | 71813,0 | 49919,0 | 1,44 | 2,0 | 10,2 | 2,3,3-Trimethylhexane |
| 4.491 | 37896 | 28363,0 | 1,34 | 1,1 | 5,4 | 2,4-dimethyl-1-Heptene |
| 6.312 | 19073 | 14016,0 | 1,36 | 0,5 | 2,7 | β-Pinene |
| 7.058 | 3356 | 4428,0 | 0,76 | 0,1 | 0,5 | 6-Ethyl-2-methyldecane |
| 7.154 | 42152 | 31130,0 | 1,35 | 1,2 | 6,0 | 3-Hexenyl acetate |
| 7.560 | 144818 | 91430,0 | 1,58 | 4,1 | 20,6 | β-ocimene |
| 7.609 | 337559 | 223214,0 | 1,51 | 9,6 | 48,1 | D-Limonene |
| 9.037 | 363160 | 223426,0 | 1,62 | 10,3 | 51,7 | Linalool |
| 10.596 | 212869 | 129103,0 | 1,65 | 6,1 | 30,3 | 4-Ethylbenzaldehyde |
| 10.799 | 192441 | 112328,0 | 1,71 | 5,5 | 27,4 | Methyl salicylate |
| 10.903 | 105265 | 62366,0 | 1,69 | 3,0 | 15,0 | Tridecane* |
| 12.141 | 6250147 | 3743405,0 | 1,67 | 178,1 | 890,6 | 1-(4-ethylphenyl)-Ethanone |
| 12.376 | 740478 | 487879,0 | 1,47 | 21,1 | 105,5 | Tetradecane |
| 13.747 | 246415 | 154404,0 | 1,6 | 7,0 | 35,1 | 1,2,3,6-Tetramethylbicyclo[2.2.2]octa-2,5-diene |
| 14.130 | 248379 | 129752 | 1,91 | 7,1 | 35,4 | α-Farnesene |
| 15.089 | 1166537 | 728048 | 1,6 | 33,2 | 166,2 | Hexadecane |
| 16.250 | 192106 | 120249 | 1,6 | 5,5 | 27,4 | Pentadecane |
| 23.366 | 223443 | 128901 | 1,73 | 6,4 | 31,8 | 2,2-Dimethyleicosane |

*Internal standard.
